# Translational and clinical development of therapeutic siRNA and ASOs: current industry practices, perspectives, and recommendations

**DOI:** 10.1093/nar/gkaf778

**Published:** 2025-09-29

**Authors:** Jesper Kammersgaard Christensen, Nicholas Colletti, Shirin Hooshfar, Rongrong(Rosa) Jiang, Carol Kuo, Bosse Lindmark, Annie Lumen, Amir S Youssef, Matthew Albertolle, Krishna C Aluri, Linzhi Chen, Jingxian Chen, Girish Chopda, Xien Yu Chua, Pedro Morais, Barent Dubois, Francis (Frank) Gibbons, Shyam Kumar Gudey, Swati Gupta, Steve Hood, Sara Humphreys, Christophe Husser, Felix Huth, Darshana Jani, Wenying Jian, Thomas Kakuda, Jeffrey Kurz, Helle Linnebjerg, Jing Liu, Sabine Lohmann, Tao Niu, Katharina Root, Sumeet Singla, Chenxiao Tang, Ravikanth Veluri, Guangnong (Sunny) Zhang, Steven Zhang, Vibha Jawa

**Affiliations:** Development ADME, Novo Nordisk, Novo Nordisk Park, Måløv 2760, Denmark; Precision Medicine, BioAnalytical & Translational Sciences, Bristol Myers Squibb, Princeton, NJ 08543, United States; Investigative ADME/Toxicology and Bioanalytical Research, Eli Lilly and Company, Indianapolis, IN 46285, United States; Drug Metabolism and Pharmacokinetics, Eisai Inc., Cambridge, MA 02140, United States; DMPK&M, Takeda Pharmaceutical, Cambridge, MA 02142, United States; Drug Metabolism and Pharmacokinetics, Research and Early Development, Cardiovascular, Renal and Metabolism, Biopharmaceuticals R&D, AstraZeneca, 431 53 Gothenburg, Sweden; Clinical Pharmacology Modeling and Simulation, Amgen Inc., South San Francisco, CA 94080, United States; Clinical Pharmacology Modeling and Simulation, GSK, Collegeville, PA 19426, United States; Development Sciences, Avidity Biosciences, San Diego, CA 92121, United States; GDDS Boston DMPK, Novo Nordisk, 33 Hayden Ave, Lexington, MA 02421, United States; Drug Metabolism and Pharmacokinetics, Boehringer Ingelheim Pharmaceuticals, Ridgefield, CT 06812, United States; Clinical Pharmacology and Pharmacometrics, Biogen, Cambridge, MA 02142, United States; GDDS Boston DMPK, Novo Nordisk, 33 Hayden Ave, Lexington, MA 02421, United States; Early Development, Alnylam Pharmaceuticals, Cambridge, MA 02142, United States; Preclinical Development, Research and Development, Bayer Pharmaceuticals, Wuppertal 42113, Germany; GDDS Boston Toxicology, Novo Nordisk, 33 Hayden Ave, Lexington, MA 02421, United States; Quantitative Pharmacology and Translational Sciences, Takeda Pharmaceuticals, Cambridge, MA 02139, United States; Bioanalytics, Moderna, Cambridge, MA 02139, United States; Development Biological Sciences, AbbVie, Irvine, CA 92612, United States; Pre-clinical Sciences, GSK Medicines Research Centre, Gunnels Wood Road, Stevenage, SG1 2NY Hertfordshire, United Kingdom; Pharmacokinetics & Drug Metabolism-Bioanalytical, Amgen Inc., South San Francisco, CA 94080, United States; Roche Pharma Research and Early Development (pRED), Roche Innovation Center, 4070 Basel, Switzerland; Pharmacokinetic Sciences, Novartis Pharma AG, 4056 Basel, Switzerland; Bioanalytics and molecular team, Moderna, Cambridge, MA 02139, United States; Bioanalytical Discovery & Development Sciences, Johnson & Johnson Innovative Medicine, Spring House, PA 19477, United States; Clinical Pharmacology & Pharmacometrics, Johnson & Johnson Innovative Medicine, San Diego, CA 92121, United States; Early Development, Alnylam Pharmaceuticals, Cambridge, MA 02142, United States; Exploratory Medicine and Pharmacology, Eli Lilly and Company, Indianapolis, IN 46285, United States; Clinical Pharmacology, Pfizer Research & Development, Groton, CT 06340, United States; Roche Pharma Research and Early Development(pRED), Roche Innovation Center Munich, Roche Diagnostics GmbH, 82377 Penzberg, Germany; Quantitative Clinical Pharmacology, Sarepta Therapeutics, Cambridge, MA 02142, United States; Pharmacokinetic Sciences, Novartis Pharma AG, 4056 Basel, Switzerland; PK Sciences, Novartis Pharmaceuticals Corporation, East Hanover, NJ 07936, United States; Clinical Pharmacology and Quantitative Pharmacology, Clinical Pharmacology & Safety Sciences, R&D, AstraZeneca, Waltham, MA 02451, United States; ADME, Eli Lilly and Company, Indianapolis, IN 46285, United States; RNAi Bioanalysis and ADME, Novo Nordisk, 65 Hayden Ave, Lexington, MA 02421, United States; Clinical Pharmacology, Novo Nordisk, 75 Hayden Ave, Lexington, MA 02421, United States; Epivax Inc, Providence, RI 02909, United States

## Abstract

RNA-based therapies, particularly small interfering RNA (siRNA) and antisense oligonucleotides (ASOs), represent a promising modality class with the potential to target previously “undruggable” proteins, and with potential for precision medicine approach. The successful development of these therapeutics relies on a comprehensive understanding of several key factors, including bioconjugation, bioanalytical techniques, biotransformation, tissue distribution, computational modeling and simulation, and clinical pharmacology. Bioconjugation strategies are essential for enhancing metabolic stability, facilitating cellular uptake, and targeting specific tissues, thereby improving efficacy and minimizing dosing. Tailored bioanalytical methods are crucial for assessing pharmacokinetics (PK) and pharmacodynamics, with particular emphasis on tissue PK in cases where plasma PK does not reflect therapeutic activity. Biotransformation and tissue distribution studies are essential, although a less comprehensive package may be adequate for well-established chemistry. Given the unique properties of oligonucleotides, computational modeling plays a critical role in predicting drug behavior in target tissues, supporting dose optimization. Clinical pharmacology for oligonucleotides is less complex than for small molecules, as they are less likely to interact with common drug metabolizing enzymes or transporter proteins. This white paper, developed by the Innovation and Quality (IQ) Consortium Nucleic Acids Working Group, consolidates industry insights and recommendations to inform best practices and regulatory guidelines, ensuring the safe and efficient development of siRNA and ASO therapies.

## Graphical Abstract

**Graphical Abstract:**
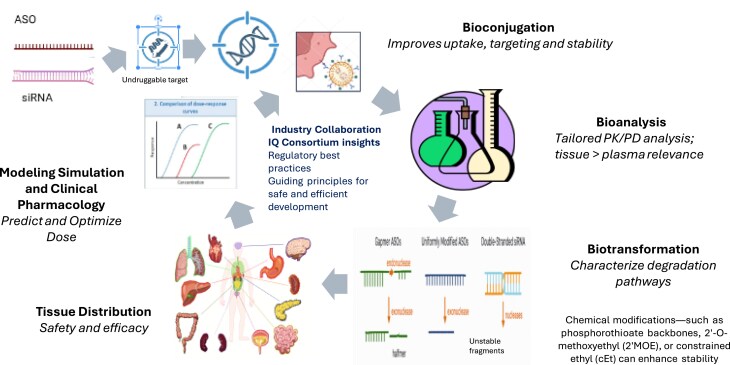

## Introduction

RNA-based therapies, including small interfering RNA (siRNA) and antisense oligonucleotides (ASOs), represent a new wave of precision medicines. Their ability to selectively modulate gene expression allows them to target diseases that were previously considered “undruggable”.

Successful development of these therapies depends on key factors such as bioconjugation (to improve delivery and stability), bioanalysis (to measure drug levels and effects), biotransformation (how the drug is metabolized), and tissue distribution. Because these drugs act in specific tissues rather than the bloodstream, understanding tissue pharmacokinetics (PK) is critical. Computational modeling can help predict this behavior and guide dose selection. These factors are discussed in detail in individual sections below.

Compared to small molecules, oligonucleotides generally have fewer safety concerns related to drug metabolism or off-target interactions. However, regulatory guidelines for these therapies are still evolving. In this white paper, the IQ Consortium Nucleic Acid Working Group shares industry insights and best practices across these key areas, with the goal of informing future regulatory frameworks and accelerating patient access to these promising treatments.

## Bioconjugation

The therapeutic potential of oligonucleotides is often hampered by limitations associated with *in vivo* delivery to target cells or tissues and their rapid degradation by nucleases. These limitations can be overcome by a variety of bioconjugation strategies in which the oligonucleotide is covalently bound, commonly via a linker, to sugars (e.g. N-acetylgalactosamine, GalNAc), lipids, peptides, proteins, polymers, and antibodies.

Bioconjugation of oligonucleotides with GalNAc is a well-established approach for targeting hepatocytes via the asialoglycoprotein receptor (ASGPR, abundantly expressed on the hepatocyte surface) for treatment of various liver diseases. GalNAc conjugation has demonstrated significant clinical success in targeting hepatocytes through the ASGPR, leading to approved therapies for various liver diseases [1]. This targeted delivery approach is also being actively explored for liver-related malignancies, such as hepatocellular carcinoma, with Alnylam’s ALN-BCAT-001 currently in a Phase 1 clinical trial (NCT06600321) evaluating its safety and efficacy in this indication. There are several GalNAc-siRNAs and one GalNAc-ASO that have been approved.

Bioconjugation with antibodies allows for targeted delivery to specific cells or tissues, increasing the oligonucleotide’s concentration at the desired site of action. This approach offers a versatile strategy for directing oligonucleotides to specific cell types or tissues by leveraging the selective binding properties of conjugated molecules [2]. For instance, antibody-oligonucleotide conjugates are being developed to target tumor-specific antigens, such as the anti-CD22 antibody conjugated to a TLR9 agonist (TAC-001), which is currently in clinical trials for advanced solid tumors and B-cell lymphoma.

Bioconjugation with peptides can be utilized to enhance cellular uptake of oligonucleotides [3]. These peptides can be designed to bind to specific receptors on cells, enabling the oligonucleotide to be internalized more efficiently.

Bioconjugation with a polymer like polyethylene glycol (PEG) is a common strategy to enhance oligonucleotide PK by increasing solubility and preventing rapid elimination [4]. One example of a PEGylated oligonucleotide in clinical development for cancer is NOX-A12 (Olaptesed Pegol), a Spiegelmer targeting CXCL12, which has been investigated in Phase 2 trials for hematological malignancies [5, 6].

Lipid conjugation can be used to improve the oligonucleotide interaction with cell membranes, promote cellular uptake through endocytosis, facilitate endosomal escape, enable targeted delivery by interacting with lipoproteins or specific receptors on the cell surface, and protect them from nuclease-mediated degradation [7].

Another approach for enhanced delivery of oligonucleotides to specific cells and tissues, and to protect them from degradation, is nanoparticle conjugation. There are both covalent and noncovalent approaches for bioconjugation of oligonucleotides with nanoparticles [8].

### IQ Consortium Working Group recommendations (IQ recommendations)

While bioconjugation enhances oligonucleotide delivery and efficacy, it is crucial to ensure that modifications do not compromise target specificity or introduce unintended immunogenicity. Balancing these factors requires rigorous preclinical evaluation. Bio-conjugated oligonucleotides need to comply with large-scale production while maintaining cost-efficiency. Development of robust and streamlined conjugation techniques is essential. Rigorous characterization of bio-conjugated oligonucleotides is vital to ensure consistency, purity, and functionality. Development of sophisticated analytical techniques to assess conjugation efficiency, target affinity, and potential immunogenicity is crucial. Regulatory agencies require detailed information on the conjugation process, characterization data, and potential safety concerns associated with the modifications. Hence a close collaboration with regulatory bodies throughout the development process is essential.

## Bioanalysis

Robust bioanalytical methods are essential for the understanding of ADME (Absorption, Distribution, Metabolism, and Excretion) properties, pharmacokinetic (PK)/pharmacodynamic (PD) correlation, mechanism of action, and the safety of therapeutics. Oligonucleotides present challenges for bioanalysis due to their high molecular weight, negative and multiple charged nature, presence of metabolites or impurities, and high nonspecific binding.

Three major assay platforms utilized for bioanalysis of oligonucleotides are chromatography, ligand-binding assay (LBA), and polymerase chain reaction (PCR) based assays. Typically, liquid chromatography mass spectrometry (LC-MS) based assays are known for their high specificity but have relatively lower sensitivity and throughput. Conversely, LBA and PCR based methods offer higher sensitivity and throughput but exhibit lower specificity. The selection of a suitable bioanalytical method is a crucial step in oligonucleotide therapeutic development and requires the careful consideration of several factors, which will be discussed in this section.

### Chromatography-based assays for bioanalysis of oligonucleotides

LC-MS is the primary platform of choice for quantitation of small molecule bioanalysis and has been increasingly utilized for oligonucleotide therapeutics such as ASOs and siRNAs [9, 10]. It provides highly specific detection based on structural information. In comparison to some of the other assay platforms such as LBA and Stem-loop (SL) RT-qPCR (Reverse transcription quantitative polymerase chain reaction), LC-MS typically is less sensitive but has the advantage of not requiring analyte-specific reagents and can differentiate between parent and metabolites. With the advancement in sample extraction techniques and instrumentation, the sensitivity achievable on LC-MS platforms is approaching sub ng/ml range. Most approved ASOs and siRNAs in the past few years have been predominantly supported by LC-MS assays for bioanalysis (Table 1).

**Table 1.** Bioanalytical platforms used for approved oligonucleotide therapeutics in clinical studies

Approval	Drug name	Type	BA platform	LLOQ	Matrix
1998	Vitravene™ (fomivirsen)	ASO	Capillary electrophoresis	NA	Vitreous humor, plasma
2013	Kynamro™ (mipomersen)	ASO	LBA	NA	Plasma, urine
2016	Defitelio™ (defibrotide)	ASO (oligo number *n*= 2–50)	LC-UV, Fluorometric	10 ug/ml, 3 ug/ml (UV) 0.2 ug/ml (Fluorometric)	Plasma, urine
2016	Exondys 51™ (eteplirsen)	ASO [phosphorodiamidate morpholino oligomer (PMO)]	LC-Fluorescence	10 ng/ml	Plasma, urine
2016	Spinraza™ (nusinersen)	ASO	LBA	NA	Plasma, cerebrospinal fluid (CSF)
2018	Tegsedi™ (inotersen)	ASO	LBA	1 ng/ml	Plasma
2018	Onpattro™ (patisiran)	Nanoparticle encapsulated siRNA	LC-Fluorescence	1 ng/ml	Plasma
2019	Givlaari™ (givosiran)	GalNAc-siRNA	Liquid chromatography coupled with high-resolution mass spectrometry (LC-HRMS), LC-MS/MS	10–20 ng/ml (Plasma) 50 ng/ml (Urine)	Plasma, urine
2019	Vyondys 53™ (golodirsen)	ASO	LC-MS/MS	10 ng/ml (Plasma) 500 ng/ml (Urine)	Plasma, urine
2020	Viltepso™ (viltolarsen)	ASO	LC-MS/MS	20 ng/ml	Plasma
2020	Oxlumo™ (lumasiran)	GalNAc-siRNA	LC-HRMS	10 ng/ml	Plasma, urine
2021	Waylivra™ (volanesorsen)*	ASO	NA	NA	NA
2021	Amondys 45™ (casimersen)	ASO	LC-MS/MS	10 ng/ml	Plasma, urine
2021	Leqvio™ (inclisiran)	GalNAc-siRNA	LC-HRMS	10 ng/ml	Plasma, urine
2022	Amvuttra™ (vutrisiran)	GalNAc-siRNA	LC-HRMS	10 ng/ml	Plasma, urine
2023	Qalsody™ (tofersen))	ASO (20mer RNA)	LBA	NA	CSF, plasma
2023	Rivfloza™ (nedosiran)	GalNAc-siRNA	LC-Fluorescence	1.0 ng/ml	Plasma, urine, dialysate
2023	Wainua™ (eplontersen)	ASO (20mer DNA)	LBA	0.129 ng/ml	Plasma

a* Only approved by EMA, not FDA.

Reference: Drugs@FDA: FDA-approved drugs (accessdata.fda.gov)

To achieve the desired sensitivity and specificity for LC-MS assays, a key element to consider is the sample extraction. Solid-phase extraction based on a weak anion exchange mechanism is increasingly utilized, with the development of commercial and generic kits. Methods can be developed relatively fast (within a few days) which provides quick access to data. However, as a generic extraction method, the efficiency of sample cleaning may still be inadequate to achieve highly sensitive assays. In addition, recovery of lipid conjugated oligonucleotides could be notably low. For enhanced sensitivity, hybridization approaches using a complementary chain probe immobilized on magnetic beads have been developed [11–15].

Liquid chromatography of oligonucleotides is particularly challenging. It requires utilization of high concentration ion pair reagents such as triethylamine and diisopropylethylamine, and organic acid such as hexafluoro isopropanol for optimal chromatographic resolution. These additives are expensive and unstable in the mobile phase; therefore, they require fresh preparation. Often, the LC column must be maintained at elevated temperatures to achieve the desired chromatographic separation and peak shape, which inevitably shortens column lifetime. Furthermore, those additives are notoriously detrimental to the mass spectrometer and could severely dampen the signals for other analytes. Therefore, the mass spectrometer used for oligonucleotide analysis should ideally be dedicated and not shared with other analytes, which adds a resource burden.

Two main mass spectrometry platforms are used for oligonucleotide bioanalysis: LC-MS/MS on a triple quadrupole and LC-HRMS on high-resolution instruments like Orbitrap or Time of Flight (TOF). Triple quadrupoles are widely used for targeted quantitation due to their high sensitivity and selectivity. However, they can face signal crosstalk issues when metabolites and parent compounds share the same nominal mass-to-charge (m/z) ratios or fragments [16]. In contrast, HRMS offers higher mass accuracy and resolution, reducing crosstalk and enabling both targeted quantitation and untargeted metabolite identification. This has led to its growing use in oligonucleotide bioanalysis, as seen in recent siRNA drug approvals (Table 1).

Quantitation on HRMS is often conducted by summing selected isotope peaks from one or more charge states to generate chromatographic peaks. On the other hand, spectra deconvolution of multiple charge states is an important step for qualitative analysis such as metabolite identification. Appropriate software for deconvolution is crucial for accurately establishing the precise masses and charge states of oligonucleotides. Different software packages offer specific features for interpreting complex mass spectra and identifying individual components within the spectra.

Another key consideration for LC-MS based assays is the use of an internal standard (IS) that is typically added at the beginning of sample extraction to track any variability in extraction recovery, injection volume, and matrix effects. For small molecules, the desired IS is a stable isotope labeled version of the analyte which shows identical physiochemical properties to the analyte but with a different molecular weight. For oligonucleotides, it is more challenging and costly to obtain such IS, although there have been reported use of IS labeled with ^34^S isotope on the backbone [17]. Alternative approaches, such as using a generic IS (unrelated sequence), sequence variants, shortened sequence, or sequence with additional nucleotides have been shown to be successful [14, 18].

As an alternative to LC-MS, LC-Fluorescence detection (LC-FD) offers a chromatography-based platform for the bioanalysis of oligonucleotides. This method involves hybridization of the analyte with a fluorescently labeled probe complementary to the antisense strand. The resulting complex is retained on anion exchange chromatography and detected via fluorescence. Typically, the probe is a peptide nucleic acid (PNA), in which the phosphate backbone is replaced by a pseudopeptide, reducing repulsive forces and enhancing complex stability. LC-FD can achieve sensitivity comparable to or better than LC-MS.

However, due to fluorescence’s inability to distinguish between complexes of parent and metabolite with the probe, extensive LC separation is required for selective detection. In cases where the major active metabolite, such as AS(n-1)3′, cannot be resolved from the parent peak, the assay can be developed as a total active method, measuring both entities as a single peak. Additionally, the PNA probe technology used in this method is patented (EP2337860B1 and US20110201006A1), and its application must be evaluated from an intellectual property (IP) standpoint.

### LBA for bioanalysis of oligonucleotides

A common method for oligonucleotide detection is LBA [19]. The analyte is captured by a binding reagent and detected using a signal-generating probe. LBA offer high sensitivity (pg/ml), minimal sample preparation, and is ideal for high-throughput assays, especially when sample volumes are limited, such as in rodent studies. However, it has drawbacks, including complex assay development, limited specificity, and a narrow dynamic range. LBA often detects the full-length oligonucleotide along with n-1 and sometimes n-2 metabolites, leading to potential cross-reactivity. It also cannot distinguish between conjugated and unconjugated forms of ASOs or siRNA, requiring careful evaluation during method development and validation for regulated studies [19].

Various formats of LBA are commonly used for oligonucleotides, including one-step, two-step, dual ligation, sandwich, noncompetitive ultrasensitive, and N-plex [20]. These methods mainly differ in their sensitivity and ability to detect metabolites. A recent technology developed by Meso-scale discovery (MSD), called N-plex, allows for femtomolar range sensitivity. This technology offers two formats, Two-probe or RNAse Protection Assay, both utilizing a SULFO-TAG label that emits light upon electrochemical stimulation for high sensitivity detection. Several oligonucleotide therapeutics have been approved utilizing LBA methods as the bioanalytical approach (Table 1).

### SL RT-qPCR (Reverse transcription quantitative polymerase chain reaction) for bioanalysis of oligonucleotides

PCR-based methods such as SL RT-qPCR, originally developed for microRNA quantification, can also be used to quantify oligonucleotides in therapeutic discovery and development, particularly when higher sensitivity is required [21]. With the SL RT-qPCR method, oligonucleotides of different mechanisms of action (e.g. siRNA, ASOs, or anti-microRNAs) can be detected with ultrahigh sensitivity (pg-fg/ml) [21]. This technique is particularly valuable for quantification of RNA-induced silencing complex (RISC)-loaded antisense strand for direct measurement of target engagement of siRNA [22, 23]. In pre-clinical studies, it has been demonstrated that siRNA levels in the RISC complex within hepatocytes are more reliable predictors of PD for GalNAc conjugated siRNAs compared to plasma PK. These findings are particularly valuable in determining effective and safe clinical dosing regimens when human liver PK profiles are unavailable [24].

SL RT-qPCR begins with a reverse transcription step using a SL primer that hybridizes to the 3′-end (typically 6–10 nucleotides) of the oligonucleotide analyte. This creates a complementary DNA (cDNA) that includes both the analyte and the SL primer sequence. The cDNA is then quantified via qPCR using specific forward and reverse primers along with a detection probe (either sequence-specific like TaqMan or a general binder like SYBR Green™). While each analyte requires a unique set of reagents, assay development is streamlined when working with panels of oligos that share sequences but differ in chemical modifications. The SL primer can be optimized to detect shortened metabolites [e.g. AS(n-1)3′], allowing the assay to estimate total active concentrations—provided the assay is fully characterized for metabolite specificity and amplification efficiency.

SL RT-qPCR offers high sensitivity, a broad dynamic range, and compatibility with high-throughput formats. However, accurate quantification may require extensive sample dilution, which can introduce variability. Due to its reliance on short 3′-end hybridization (6–10 base pairs), the method may have lower specificity compared to techniques like LC-MS. While promising for drug discovery applications, SL RT-qPCR has not yet been widely adopted for regulated studies. Barriers include the absence of regulatory guidance for qPCR-based bioanalytical methods, limited industry experience with PCR platforms, challenges in data processing, and integration with Laboratory Information Management Systems.

Recently, digital droplet variations of the SL RT-qPCR assay have been suggested to achieve absolute quantification of oligonucleotides [25]. In this format, the PCR reaction is partitioned into thousands of droplets (>10 000), in which each of them has an average of a single cDNA copy. Digital dropletSL RT-qPCR could have some advantages, such as a higher tolerance for inhibitors of qPCR reactions (due to sample partitioning in single droplets) and no need for a standard curve. However, the digital droplet RT-qPCR can have a lower dynamic range than standard RT-qPCR.

### IQ recommendations for bioanalysis

Several quantitative bioanalysis techniques for oligonucleotides have been established (Table 2). The selection of bioanalytical approach should be case-specific, considering factors like sensitivity, method limitations, metabolism, reagent availability, program stage, and regulatory needs. LC-MS offers higher specificity and quicker assay development as it does not require sequence-tailored reagent. On the other hand, LBA and qPCR offer higher sensitivity and throughput. When both sensitivity and specificity are crucial, hybridization LC-MS can achieve sub-ng/ml sensitivity [15, 18].

**Table 2.** Comparison of major assay platforms used for bioanalysis of oligonucleotides

Platform	LC-MS	LC-FD	LBA	SL RT-qPCR
Sample preparation	Liquid-liquid extraction, solid phase extraction, or hybridization	Hybridization	Dilution and hybridization	Dilution, reverse transcription, and amplification
Sensitivity	Relatively low Typically, >1 ng/ml, Sub ng/ml for hybridization	Medium 0.1–1 ng/ml	High Typically sub ng/ml Can be at pg/ml	Highest pg/ml
Specificity	High	Medium	Low	Low
Metabolite detection	Yes	Can be optimized	Can be optimized	Can be optimized
Custom reagent	IS, probe for hybridization	Probe	Probe	Primer and probe
Assay development	Fast, days-weeks	Fast, days-weeks	Slow, weeks-months	Medium, weeks
Throughput	Low	Low	High	High
Challenges	Chromatography, ionization, sensitivity, ruggedness	Metabolite separation, specificity, IP consideration	Probe design, specificity	Primer design, specificity, assay ruggedness
Regulatory BA	Yes	Yes	Yes	Not routine
Key references	[9, 12, 14, 15, 18, 28]	[29]	[19, 30]	[21, 31]

LC-MS has the advantage of being able to elucidate the drug metabolite profile and quantify parent/metabolite specifically. In the case when LBA or qPCR is being used in the advanced stage of a program, the method should be fully characterized for the specificity against the active metabolite(s).

As oligonucleotide modalities evolve, such as antibody or peptide oligonucleotide conjugates, bioanalytical strategies may need to be adapted to quantify different components of the molecules. [26, 27]. Ultimately, no single assay fits all scenarios and choosing a strategy involves multiple factors. It is advisable to maintain the same assay platform throughout studies. If changes occur, it is recommended to provide scientific justification and compare data from different platforms to evaluate impacts.

## Biotransformation

Oligonucleotides are primarily metabolized by endo- and exonucleases, which are widely expressed in the body. Unmodified oligonucleotides degrade rapidly (within minutes), so chemical modifications—such as phosphorothioate backbones, 2′-O-methoxyethyl (2′MOE), or constrained ethyl (cEt)—are used to enhance stability. For gapmer ASOs, metabolism starts with endonuclease cleavage in the central gap, producing “halfmers”, followed by exonuclease digestion [32, 33]. In contrast, uniformly modified ASOs, like splice-switching oligonucleotides (SSOs), are mainly degraded by exonucleases and are generally more stable. Double-stranded siRNAs are more stable than single-stranded ASOs but remain susceptible to nuclease degradation [34]. Unlike small molecules, oligonucleotide metabolism shows greater cross-species similarity, since it relies on nucleases rather than enzymes like cytochrome P450s [24].

As summarized in the earlier sections, ASOs and siRNAs are commonly conjugated with linkers presenting three GalNAc ligands for targeting hepatocytes via the ASGPR. GalNAc conjugates are relatively labile, and exhibit rapid loss of one, two, or all three sugar moieties in hepatocytes, a process mediated by a lysosomal enzyme N-acetyl-β-glucosaminidase. Biotransformation pathways of a trishexylamino linker in a GalNAc conjugated ASO has been studied in detail, where 14 novel linker derived metabolites were identified [35]. Metabolism of the linker, via cleavage of its amide bonds, has also been reported for a GalNAc-siRNA [24]. A similar strategy utilising lipids, peptides and antibodies, as discussed earlier, is being explored as conjugates to achieve delivery of oligonucleotides to tissues beyond the liver. Biotransformation of these novel conjugates could be diverse and depend on the characteristics of both the conjugated moiety and the associated linker.

Based on the combined knowledge of this working group, truncated gapmer ASO metabolites are generally inactive. In contrast, significant focus has been directed toward pharmacologically active metabolites of the antisense strands in siRNAs. siRNA metabolism predominantly consists of sequentially chain-shortened segments, with AS(n-1)3′ (antisense strand with loss of one nucleotide from the 3′-terminus) often being pharmacologically active. Further truncated metabolites typically show progressively reduced activities. However, loss of up to five nucleotides from the 3′-terminus [i.e. AS(n-5)3′] has been reported without loss of activity [24].

The AS(n-1)3′ metabolite has been shown to retain pharmacological activity equal to that of the parent compound for both the GalNAc-conjugated siRNAs givosiran [36] (FDA NDA 212194) and lumasiran [37] (FDA NDA 214103). Additionally, the deamination of terminal adenosine to inosine has resulted in pharmacologically active metabolites for both the single-stranded RNA reverser [13] and GalNAc-siRNA lumasiran. Notably, this deamination pathway is not conserved across species and appears to be specific to humans and nonhuman primates (NHPs). Deamination has also been reported in ASOs [38, 39].

Compared to the parent full-length oligonucleotide, truncated metabolites do not pose an additional risk for off-target effects, as their affinity for off-target mRNAs via Watson–Crick base pairing is expected to be lower than that of the parent compound [40]. However, there is a theoretical concern that oligonucleotides catabolized into monomer nucleotides might exhibit toxicity similar to that observed with nucleotide/nucleoside-based anticancer and antiviral drugs. For example, the nucleoside analogue fialuridine, once used to treat chronic hepatitis B virus infection, is associated with severe hepatotoxicity due to mitochondrial damage [41].

Safety evaluations related to metabolism of 2′-fluoro (2′-F) containing GalNAc-siRNAs concluded that exposure to 2′-F monomer metabolites was low and transient in both rats and humans. The data support the safe application of the 2′-F modification in metabolically stabilized therapeutic GalNAc-siRNAs, particularly when enhanced stabilization chemistry is employed [42]. Furthermore, the 2′OMe-uridine modification in siRNAs has been demonstrated to be safe, undergoing demethylation to uridine and following normal endogenous pathways [43]. Similarly, 2′OMe-cytidine is metabolized to uridine and cytidine triphosphate and is therefore considered a safe motif in oligonucleotide therapeutics [44].

In vivo studies of oligonucleotide metabolism typically use plasma, urine, and tissues where the drug accumulates—such as the liver, kidney, or the intended target organ in preclinical models. Plasma often shows low levels of metabolites because the parent oligonucleotide is rapidly taken up into tissues. In contrast, tissues—especially the liver—tend to have higher metabolite levels, making them a better matrix for evaluating key metabolic pathways. Analyzing tissue homogenates from preclinical species can also provide insights into metabolism at the target site, including tissue-specific transformations like deamination.

The types of metabolites detected can differ depending on the matrix. Plasma usually contains metabolites that are structurally close to the full-length parent compound, while urine is typically enriched with smaller metabolites. This is because shorter fragments have lower protein binding and are more easily eliminated through the kidneys.

Unlike small molecules, where ADME studies using radiolabeled compounds are standard, this approach is less useful for oligonucleotides. Radiolabeling for oligonucleotides is more relevant to tissue distribution studies and is discussed separately. Instead, LC-HRMS (liquid chromatography coupled with high-resolution mass spectrometry) has become the primary method for metabolite profiling of oligonucleotides due to its sensitivity and ability to detect a wide range of metabolite species (as discussed in the ‘Bioanalysis’ section).

To support the ethical principles of the 3Rs—Replacement, Reduction, and Refinement of animal use—various *in vitro* models have been developed as alternatives to *in vivo* studies for assessing oligonucleotide metabolism. These include: Serum incubations [45] (https://www.accessdata.fda.gov/drugsatfda_docs/nda/2022/215515Orig1s000TOC.cfm); Human hepatocyte co-culture model (HepatoPac^®^) [37] (https://www.accessdata.fda.gov/drugsatfda_docs/nda/2020/214103Orig1s000TOC.cfm); Liver S9 fractions [46] (https://www.ema.europa.eu/en/documents/assessment-report/leqvio-epar-public-assessment-report_en.pdf); Hepatic microsomes [47] (https://www.accessdata.fda.gov/drugsatfda_docs/nda/2021/213026Orig1s000TOC.cfm); and Liver homogenates [48] (https://www.pmda.go.jp/files/000236289.pdf).

These *in vitro* systems have shown good correlation with metabolite profiles observed in both preclinical species and humans. However, it is important to note that metabolism of oligonucleotides can vary widely depending on their chemical structure, nucleotide sequence, conjugates or linkers, and route of administration. Therefore, no single *in vitro* model can fully predict the *in vivo* metabolism for every oligonucleotide therapeutic.

### Regulatory landscape

Although oligonucleotides are classified as small molecule drugs by regulatory agencies, they share several characteristics with peptides and biologics. As a result, certain deviations from traditional small molecule expectations may be acceptable for oligonucleotide therapeutics. For instance, Japan’s *Guideline for Preclinical Safety Assessment of Oligonucleotide Therapeutics* [49] (https://www.pmda.go.jp/files/000236289.pdf) states that naturally occurring nucleic acid components formed via nuclease degradation raise no particular safety concerns. However, metabolites containing chemically modified moieties must be evaluated in accordance with ICH M3(R2) [50] (https://www.ema.europa.eu/en/documents/scientific-guideline/ich-guideline-m3r2-non-clinical-safety-studies-conduct-human-clinical-trials-and-marketing-authorisation-pharmaceuticals-step-5_en.pdf), as with conventional chemical entities.

ICH M3(R2) [50] recommends conducting *in vitro* metabolism studies before the first human dose. It also requires that metabolite exposures in human plasma be assessed—potentially in toxicology studies—prior to Phase III trials. For innovative modalities such as siRNAs, ICH M3(R2) allows flexibility where studies may be abbreviated, deferred, or omitted. This flexibility is reflected in the variable extent of biotransformation data included in approved oligonucleotide submissions, which do not always fully align with ICH expectations.

The FDA’s draft guidance on *Clinical Pharmacology Considerations for Human Radiolabeled Mass Balance Studies* [51] (https://www.fda.gov/regulatory-information/search-fda-guidance-documents/clinical-pharmacology-considerations-human-radiolabeled-mass-balance-studies) suggests that a mass balance study may not be necessary for oligonucleotide therapeutics if their metabolism and excretion are well understood through nonclinical ADME data. Similarly, the guidance on *Clinical Pharmacology Considerations for the Development of Oligonucleotide Therapeutics* [52] (https://www.fda.gov/regulatory-information/search-fda-guidance-documents/clinical-pharmacology-considerations-development-oligonucleotide-therapeutics) outlines bioanalytical requirements for metabolite characterization. The draft guidance on *Nonclinical Safety Assessment of Oligonucleotide-Based Therapeutics* [53] further emphasizes assessing predicted metabolites for off-target hybridization, conducting *in vitro* assessments, and comparing human and animal metabolite profiles *in vivo*.

While existing guidelines offer useful direction, they contain inconsistencies and provide limited clarity on the specific biotransformation studies needed—or those that can be omitted—for a successful NDA/MAA. To address these gaps, this working group has developed industry-aligned recommendations to help standardize expectations and streamline development pathways for oligonucleotide-based therapeutics.

### Approved biotransformation packages

In order to get more information on regulatory precedence for metabolism of oligonucleotides, a review of the FDA-approved ASO and siRNA drugs was performed (Table 3). Metabolism studies have been performed for all approved oligonucleotide therapies. Most of siRNA metabolism packages (four out of six) include a combination of *in vitro* studies, as well as *in vivo* studies with and without radiolabeling. Conversely, this level of comprehensiveness is not found in any of the ASO metabolism packages (11 in total).

**Table 3.** Biotransformation studies conducted for approved ASOs and siRNAs^1^

Drug name (API)	FDA approval year	Format	*In vitro* studies (system, species)	*In vivo* studies with nonradiolabeled compound (species, matrices)	*In vivo* studies with radiolabeled compound (species, matrices)
Vitravene^TM^ (fomivirsen)	1998	ASO	ND	Monkey (plasma, liver, kidney)	[^14^C] rabbit (vitreous, retina)
Kynamro^TM^ (mipomersen)	2013	ASO gapmer	Liver microsomes (human)	Mouse, rat, and monkey (liver, kidney); rat, monkey, and human (urine)	ND
Exondys 51^TM^(eteplirsen)	2016	ASO-PMO SSO	Liver microsomes (mouse, rat, monkey, human)	ND	ND
Spinraza^TM^ (nusinersen)	2016	ASO SSO	ND	Mouse (liver), monkey (CSF, brain cortex, lumbar spinal cord, liver, kidney, plasma), human (CSF, plasma, urine)	ND
Onpattro^TM^ (patisiran)	2018	LNP-formulated siRNA	Serum and liver S9 (mouse, rat, monkey, human)	Rat (urine)	ND
Tegsedi^TM^ (inotersen)	2018	ASO gapmer	ND	Mouse (plasma, urine, liver, kidney), monkey (plasma, liver, kidney), human (plasma, urine)	[^3^H] rat (plasma, urine, faeces, liver, kidney)
Waylivra^TM^ (volanesorsen)	20 19^2^	ASO gapmer	ND	Mouse and monkey (plasma, urine, liver, kidney), human (plasma, urine)	[^3^H] rat (plasma, urine, liver, kidney)
Givlaari^TM^ (givosiran)	2019	GalNAc-conjugated siRNA	Liver S9 (mouse, rat, monkey, human)	Human (plasma, urine)	[^3^H] rat (plasma, urine, bile, faeces)
Vyondys 53^TM^ (golodirsen)	2019	ASO-PMO SSO	ND	ND	[^14^C] human (plasma, urine)
Viltepso^TM^ (viltolarsen)	2020	ASO-PMO SSO	ND	Human (plasma, urine)	ND
Oxlumo^TM^ (lumasiran)	2020	GalNAc-conjugated siRNA	Serum and liver S9 (mouse, rat, monkey, human)	Rat and monkey (plasma, liver), human (plasma, urine)	ND
mondys 45^TM^ (casimersen)	2021	ASO-PMO SSO	Liver microsomes (mouse, rat, monkey, human)	ND	[^14^C] human (plasma, urine)
Leqvio^TM^ (inclisiran)	2021	GalNAc-conjugated siRNA	Serum and liver S9 (mouse, rat, monkey, human)	Rat, monkey, and human (plasma)	[^14^C] rat (plasma, urine, bile, feces, liver, kidney, injection site), [^14^C] monkey (plasma, urine, feces, liver, kidney)
Amvuttra™ (vutrisiran)	2022	GalNAc-conjugated siRNA	Serum and liver S9 (mouse, rat, monkey, human)	Human (plasma)	[^3^H] rat (plasma, liver)
Qalsody^TM^ (tofersen)	2023	ASO gapmer	Hepatocytes (human)	ND	ND
Rivfloza^TM^ (nedosiran)	2023	GalNAc-conjugated siRNA	Hepatocytes (human)	Mice (plasma, urine, liver), monkey, and human (plasma, urine)	[^3^H] mouse (plasma, urine, feces, bile)
Wainua^TM^ (eplontersen)	2023	GalNAc-conjugated ASO gapmer	ND	Human (plasma)	[^3^H] rat and monkey (plasma, liver, kidney)

API, active pharmaceutical ingredient; ASO, antisense oligonucleotide; GalNAc, N-acetylgalactosamine; LNP, lipid nanoparticle; ND, no studies disclosed; PMO, phosphorodiamidate morpholino oligomer; siRNA, small interfering ribonucleic acid; SSO, splice-switching oligonucleotide.

1: Information source: published NDA/MAA review documents.

2: Not approved by FDA, though approved by EMA in 2019.

### Timing of biotransformation studies and regulatory feedback

The approaches to conducting metabolism studies during the development of GalNAc conjugated ASOs and siRNAs, including their timing, and whether they involve *in vitro* or *in vivo* methods in rodents, nonrodents, or humans, differ among companies within the IQ consortium. These divergent approaches to metabolism studies may stem from the previously mentioned limited regulatory guidance for oligonucleotide therapies. For instance, one company postponed biotransformation studies until after the commencement of phase 1 trials, while another waived *in vitro* studies altogether during development. Additionally, a third company chose not to perform *in vivo* human biotransformation studies in plasma. Despite these varied strategies, none of these companies have faced regulatory challenges from health authorities like the FDA or EMA in the early stages of clinical development. Future filings and combined experience will need to be shared by sponsors to understand if these reduced metabolism packages are sufficient for NDA filing.

### IQ recommendations for biotransformation

Health authority guidance on biotransformation studies for oligonucleotide therapeutics is insufficient, due to lack of clarity. This working group provides insights and recommendations based on experience in oligonucleotide ADME.

- *In vitro* models: Emphasize the use of *in vitro* models to study oligonucleotide metabolism, aiming to reduce reliance on *in vivo* studies while adhering to the 3Rs principle. There is no one-size-fits all *in vitro* system, and therefore a platform-centric, fit-for-purpose approach is suggested. *In vitro* characterization could be of value to study metabolism of novel conjugates and linkers before first-in-human (FIH) studies.
- Tissue relevance: For oligonucleotides with established chemistries, liver is the key matrix for biotransformation studies. For drugs targeting tissues beyond liver, biotransformation studies in the intended target tissue are likely of importance.
- Metabolite profiling: Conduct metabolite profiling after repeat dosing and utilize tissue samples after necroscopy in toxicology studies to comply with 3R.
- Human studies: In humans, where tissue biopsies are rarely available, relevant *in vitro* systems can provide insights into human metabolism. In vivo analysis of both plasma and urine could offer a more complete picture of oligonucleotide metabolism.
- MIST evaluation: The FDA MIST guidance [54] is not recommended to be applied for oligonucleotide therapeutics with well-established chemistries for these reasons: (i) low risk of human disproportionate metabolites; (ii) much higher metabolite burden in tissue than plasma, and metabolite profiles in plasma do not necessarily reflect those in tissue [13]; (iii) there are no examples of toxic metabolites of oligonucleotides to the best of our knowledge.
- Radiolabelling: The value of biotransformation studies using radiolabelled oligonucleotides is limited for established oligonucleotide platforms, as predictable metabolites can be assessed without it. LC-MS and synthesized metabolite standards can effectively support these studies.
- Novel conjugates/linkers: For new conjugates or linkers, evaluate their routes of metabolism and elimination. If they contain small molecule-like structures, demonstrate coverage of the human therapeutic plasma exposure of cleaved conjugate/linker and related metabolites in toxicology species. A study with radiolabelled compound, with the radiolabel in a stable position in the linker/conjugate, might be appropriate.
- Active metabolites: Follow the IQ Consortium guidelines regarding active metabolites [53]. Monitoring is unnecessary if the pharmacological activity index (PAI) of a metabolite is below 25%. Consider the total PAI for multiple active metabolites.

## Tissue distribution

Tissue distribution studies in preclinical development are important for confirming delivery of oligonucleotide therapeutics to both target and nontarget tissues, establishing their biodistribution profiles, and assessing sufficient exposure levels for pharmacologic activity. These studies also help identify tissues with high drug accumulation that may present toxicologic risks. Regulatory guidance documents [50, 52, 55, 56] (https://www.ema.europa.eu/en/documents/scientific-guideline/ich-guideline-m3r2-non-clinical-safety-studies-conduct-human-clinical-trials-and-marketing-authorisation-pharmaceuticals-step-5_en.pdf), (https://www.fda.gov/regulatory-information/search-fda-guidance-documents/clinical-pharmacology-considerations-development-oligonucleotide-therapeutics), (https://www.database.ich.org/sites/default/files/S6_R1_Guideline_0.pdf), (https://www.fda.gov/media/183496/download) acknowledge the value of such studies—including for oligonucleotides—but do not require them as part of the development process [56] (https://www.fda.gov/media/183496/download).

In early development, tissue distribution studies of ASOs and siRNAs are commonly conducted in rodents and NHPs, often outside of GLP compliance. These studies typically rely on nonradiolabeled bioanalytical methods that detect the active (antisense) strand, offering insight into tissues with high exposure for targeted GLP toxicology assessments. Table 2 outlines the bioanalytical methods used across sponsors. For well-characterized platforms like GalNAc-conjugated oligonucleotides, such approaches are usually sufficient, and in some cases, extensive tissue distribution studies may no longer be necessary due to the predictable biodistribution of these molecules.

For newer platforms, such as ligand-conjugates, local delivery systems, or novel chemical modifications, more comprehensive preclinical tissue distribution studies may be necessary. Quantitative whole-body autoradiography (QWBA) with phosphor imaging of frozen sections provides high-resolution (50–100 μm) images of drug distribution, throughout the body, and associated tissue concentrations [57]. Micro autoradiography can offer cellular-level resolution when needed for specific tissues. Additionally, real-time imaging of radiolabeled molecules can be conducted noninvasively using positron emission tomography [58] and single photon emission computed tomography (SPECT) [59] to monitor changes in tissue distribution over time. However, these imaging methods typically have lower resolution than QWBA and often involve radiolabeling techniques that alter the molecule’s structure.

### Considerations for radiolabeling oligonucleotides

Radiolabeled oligonucleotides are used in studies of tissue distribution, mass balance, metabolite profiling, and identification. Key considerations include selecting a suitable isotope that minimizes structural changes—ideally replacing a native atom (e.g. ^1H with ^3H). Isotope choice impacts labeling efficiency, specific activity, and dose requirements. For example, ^14C’s low specific activity may require higher doses, while isotopes like ^35S (half-life ∼87.5 days) need careful timing to account for decay. Practical factors like synthesis complexity, cost, and waste disposal are also important.

Label placement is critical. Single-site internal labeling is preferred to maintain consistent specific activity and avoid label loss from exonuclease cleavage, unlike 5′ or 3′ end-labeling. Stable positions like the C5′ of the sugar (for ^3H) are favored, while unstable sites like the C8 of adenine or guanine are avoided due to tritium exchange. For ligand-conjugated oligonucleotides, the label can be placed on the ligand (e.g. lipid) to monitor its fate.

However, tracking all metabolites is challenging if the radiolabel is lost in cleavage products. This can be mitigated by labeling segments containing novel chemical groups (see Table 4) to better follow their metabolic pathways. Detailed recommendations on radiolabeling strategies are discussed here [33].

**Table 4.** Components in approved ASOs and siRNAs

	Clinically evaluated	Not clinically evaluated
Chemical modifications: sugar/base	2′O-methoxy, 2′-F, 2′-O-methoxyethyl (2′MOE), 5-methyl pyrimidine	Sugar/base modifications that have not previously been used in approved oligonucleotides
Chemical modifications: backbone	Phosphorothioate (PS), PMO	Backbone modifications that have not previously been used in approved oligonucleotides
Delivery method	Gymnotic delivery,^1^ GalNAc-conjugated with noncleavable linker	NonGalNAc ligand conjugates, cleavable linkers or linker chemistry that has not been utilized before in approved oligonucleotides
Target tissue/cell type	ASGPR-mediated hepatic uptake	Nonhepatic tissues
Cellular uptake method	ASGPR-mediated hepatocyte uptake	Other receptor mediated uptake

1: Delivery to cells in the absence of carrier or conjugation

### Regulatory landscape and approved packages

There are limited regulatory guidelines on the ADME properties of oligonucleotides. The PMDA’s 2020 guideline [49] (https://www.pmda.go.jp/files/000236289.pdf) addresses preclinical safety and mentions metabolites but not tissue distribution. In 2024, the FDA issued guidance on clinical pharmacology for oligonucleotide therapeutics [52] (https://www.fda.gov/regulatory-information/search-fda-guidance-documents/clinical-pharmacology-considerations-development-oligonucleotide-therapeutics), highlighting that chemistry (e.g. backbone modifications, conjugation), drug target, plasma protein binding, and route of administration are key factors influencing tissue distribution, particularly to the liver, kidneys, and other tissues.

A review of FDA-approved ASOs and siRNAs (Table 5) shows that tissue distribution studies were conducted for all approved drugs, typically using radiolabeled compounds, except for nusinersen. About half employed QWBA, while others measured radioactivity in selected tissues. Fomivirsen [60, 61] (https://www.accessdata.fda.gov/drugsatfda_docs/nda/98/20961_Vitravene_pharmr.pdf, https://www.accessdata.fda.gov/drugsatfda_docs/nda/98/20961_Vitravene.cfm) and tofersen [62] (https://www.accessdata.fda.gov/drugsatfda_docs/nda/2023/215887Orig1s000OEList.pdf), administered via intravitreal and intrathecal routes respectively, showed localized distribution (eye and CNS), with limited systemic exposure. Generally, for ASOs, the liver and kidney showed the highest exposure, with notable distribution to the spleen and bone marrow. For siRNAs [all GalNAc-conjugated except lipid nanoparticle (LNP)-encapsulated patisiran], highest exposure was in the liver, followed by the kidney, lymph nodes, GI tract, and bladder.

**Table 5.** Tissue distribution studies conducted for approved ASOs and siRNAs^1^

Drug name (API)	FDA approval year	Format	Species	Nuclide	Dose (mg/kg) and route of administration	Tissue with highest exposures
Vitravene™ (fomivirsen)	1998	ASO	Rabbit Monkey	^14^C ^14^C	66 μg intravitreal	Retina, iris; systemic exposure: minimal (below LOQ) No QWBA conducted
Kynamro™ (mipomersen)	2013	ASO	Mouse, Rat	^3^H	5 IV	kidney (especially cortex), liver, spleen, lymph nodes, thyroid/parathyroid, stomach and bone marrow No QWBA conducted
Exondys 51™ (eteplirsen)	2016	PMO-ASO	Mouse	^14^C	120 IV	Kidney, skeletal muscles QWBA conducted
Spinraza™ (nusinersen)	2016	ASO	Monkey	None	1 IT and IV	Central nervous system (CNS), plasma, peripheral tissue (skeletal muscle liver, kidney), fat, bone marrow, spleen No QWBA conducted
Onpattro™ (patisiran)	2018	LNP-formulated siRNA	Rat	^14^C-Dlin-MC3-DMA^2^	0.3 IV	Liver QWBA with radiolabeled LNP lipid
Tegsedi™ (inotersen)	2018	ASO	Rat	^3^H	25 SC	Liver, kidney, broad tissues QWBA conducted Liver, kidney
Waylivra™ (volanesorsen)	20 19^3^	ASO	Rat	^3^H		QWBA conducted
Givlaari™ (givosiran)	2019	GalNAc-conjugated siRNA	Rat	^3^H	10 SC	Liver, lymph nodes, urinary bladder, thoracic duct, kidneys QWBA conducted
Vyondys 53™ (golodirsen)	2019	PMO-ASO	Mouse	^14^C	120 IV	Kidney, all tissue except CNS No QWBA conducted
Viltepso™ (viltolarsen)	2020	PMO-ASO	Mouse Monkey	^14^C ^14^C	20 IV 20 IV	Kidney, skeletal muscle No QWBA conducted
Oxlumo™ (lumasiran)	2020	GalNAc-conjugated siRNA	Rat	^14^C	5 or 10 SC	Dose site, liver, kidney QWBA conducted
Amondys 45™ (casimersen)	2021	PMO-ASO	Mouse	^14^C	120 IV	Kidney, skeletal muscle No QWBA conducted
Leqvio™ (inclisiran)	2021	GalNAc-conjugated siRNA	Rat Monkey	^14^C	65 SC 20 SC	Dose site, liver, kidney QWBA conducted Dose site, liver, kidney, lymph nodes
Amvuttra™ (vutrisiran)	2022	GalNAc-conjugated siRNA	Rat	^3^H	3 SC	Liver, lymph nodes, thoracic lymph duct, kidneys, GI (gastrointestinal ) tract QWBA conducted
Qalsody™ (tofersen)	2023	ASO	Human	^99m^Tc	100 mg IT	SPECT (not QWBA)
Rivfloza™ (nedosiran)	2023	GalNAc-conjugated siRNA	Mouse	^3^H	5 SC	Liver, urinary bladder QWBA conducted
Wainua™ (eplontersen)	2023	GalNAc-conjugated ASO	Rat	^3^H	2 or 10 SC	Liver, kidney QWBA conducted

API, active pharmaceutical ingredient; CNS, central nervous system; IT, intrathecal; IV, intravenous; LOQ, limit of quantification; LNP, lipid nanoparticle; PMO, phosphorodiamidate morpholino oligomer; PO, per oral; SC, subcutaneous; SPECT, single-photon-emission computer tomography.

1: Information source: published NDA/MAA review documents.

2: The radiolabel was placed in the ionizable lipid Dlin-MC3-DMA, an excipient of the LNP (and not in the oligonucleotide).

3: Not approved by FDA, though approved by EMA in 2019.

Among IQ Nucleic Acid Working Group companies, preclinical tissue distribution studies for GalNAc-conjugated oligonucleotides typically focus on the liver and kidney using well-established chemistries (Table 4). Two companies follow this with broader studies in phase 2. Most are moving toward nonradiolabeled approaches for GalNAc compounds. Regulatory feedback has not raised major concerns on these strategies.

For oligonucleotides with novel components (Table 4), most companies plan or consider using radiolabeled studies case by case, though at least one proceeds without it. The majority conduct tissue distribution studies before FIH, while some wait until phase 2. However, many companies have not yet sought regulatory feedback on these plans.

### IQ recommendations for tissue distribution

An understanding of the tissue distribution of a therapeutic oligonucleotide candidate is required to predict on and off target pharmacology and to provide quantitative data for the first in human dose predictions and safety margins. Furthermore, since the tissue distribution of different types of ASO and siRNA molecules is driven predominantly by their chemistry and conjugation, it is typically sequence independent and thus “class rules” can be established.

This understanding has allowed new molecules to be classified whether they are structurally similar to previously approved molecules, or they have a combination of chemical modifications or targeting moieties that have not been clinically evaluated yet.

**Figure 1.**
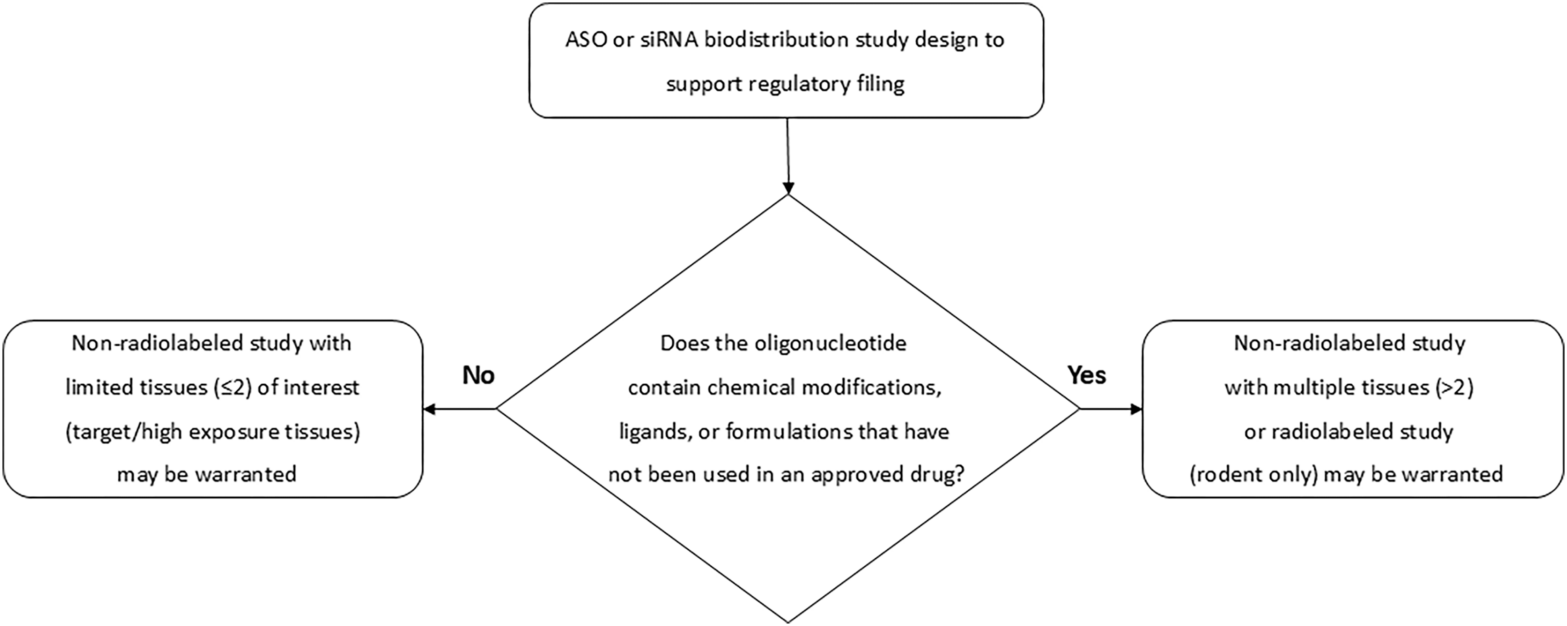
Industry recommendation on design of ASO and siRNA tissue distribution studies to support regulatory filing.

A “class rules” approach has been endorsed by the regulatory authorities and highlighted in white papers [63] and formal guidance documents [51, 56, 64, 65] (https://www.fda.gov/regulatory-information/search-fda-guidance-documents/clinical-pharmacology-considerations-human-radiolabeled-mass-balance-studies, https://www.fda.gov/media/183496/download, https://www.fda.gov/regulatory-information/search-fda-guidance-documents/development-and-licensure-vaccines-prevent-covid-19, https://www.fda.gov/media/178938/download). This regulatory awareness has resulted in the acceptance of citing literature data from similar molecules as an argument for waiving comprehensive tissue distribution packages, especially if a subset of known tissues of exposure is sampled and drug concentrations measured. Conversely, for oligonucleotide candidates with unprecedented novel configurations, it is less likely that literature or filing data could be used as a surrogate to perform the complete package. The classification of oligonucleotides into two classes is outlined in Fig. 1 and further detailed in Table 4.

Precedented regulatory filing package: In cases where regulatory precedence exists for oligonucleotides of a similar class, tissue distribution studies are considered confirmatory rather than exploratory, so evaluating exposure in minimal tissues (≤2) of interest (including tissues expressing target receptor and high exposure tissues such as liver and kidney) using nonradioactive bioanalytical methods is likely appropriate. Rodents are preferred for tissue distribution studies in most cases. Dedicated monkey tissue distribution studies are not recommended; however, with careful planning, sparse confirmatory monkey tissue distribution data may be gleaned from toxicology study animals.

Unprecedented regulatory filing package (i.e. containing novel components): Where novel nucleotide chemical modifications, linker chemistries or targeting ligands are used, a more comprehensive tissue sampling protocol is recommended to ensure the full tissue distribution profile is captured. This is especially warranted for targeting outside of the liver and kidney and for alternative dose routes (intrathecal, oral, inhaled). This work can be performed as a stand-alone tissue distribution study, or as part of a toxicology study, with nonradioactive bioanalytical methods, or a stand-alone study utilizing radioactive-labeled molecules (preference for ^3^H). When radiolabeled studies are deemed necessary, their utility can be enhanced by including mass balance objectives in the protocol, especially in situations where the mass balance is not clear, or where data from nonradioactive studies are insufficient.

The characterization of the tissue distribution of an oligonucleotide candidate is a cornerstone of a regulatory package and is expected by the regulatory authorities. The volume of de-novo data required for this package will be dictated by the nature of chemistry and the body of literature supporting it. Thus, the Nucleic Acid Working Group strongly recommends that a tissue distribution package is tailored to the familiarity or novelty of chemistry and targeting components of the oligonucleotide candidate. Furthermore, the working group encourages the publication of such data to improve collective knowledge and the expansion of the precedented design space.

## Computational modeling and simulation

Oligonucleotides (ASOs, siRNAs) are effective for modulation of gene expression where tissue PK, not plasma PK, primarily determines PD, resulting in less frequent and more convenient dosing schedules for patients. This is due to the temporal disconnect observed between the plasma PK half-lives (short, typically in hours), the tissue PK half-lives (long, typically in days to weeks), and PD or target engagement duration (long, in days to weeks) in these modalities, as highlighted for GalNAc-siRNAs. These unique PK/PD relationships require computational modeling and simulation (M&S) to bridge plasma PK, tissue PK, and PD, integrating modality-specific mechanisms for tissue distribution and target engagement. Human tissue PK data are often not readily available, emphasizing the importance of computational methods using nonclinical data for predicting clinical PK/PD to guide clinical dose selection and optimization. Traditional methods like allometric scaling and indirect-response PK/PD modeling have been successfully used in oligonucleotide development, while more mechanistic strategies like PBPK modeling are being increasingly adopted when appropriate.

The following sections summarize best practices and recommendations for M&S approaches for these modalities from expert insights across 10 IQ companies (Table 6) and provide an overview of PK/PD modeling strategies applied for approved oligonucleotide therapies (Table 7). We also discuss the limitations and knowledge gaps in developing and applying computational M&S tools to support oligonucleotides drug discovery and development.

**Table 6.** Current practice and IQ recommendations for M&S strategies and applications for siRNA and ASO at different stages of drug development

M&S applications	Current practice	IQ recommendations
Translation of PK and PD from preclinical species to humans		
Human PK prediction	BW-based allometric scaling approach widely adopted Semi-PBPK model used for intrathecally administrated ASO (i.e. NHP CSF/brain PBPK model)	Mechanistic PK models are encouraged but not necessarily required: Allometric scaling approach generally work well Bottom-up PBPK models are recommended when key ADME elements of siRNA/ASOs are available, or the following prediction outcomes are desirable: Human tissue concentration profiles PK profiles in specific patient populations Non-systemically administered oligonucleotides
Translational PK/PD	Indirect response model widely used Target tissue (not plasma) drug concentration as PD driver Kinetic-PD models to describe dose response versus time using biomarker and/or PD endpoints, when tissue concentration data are unavailable Additional effect compartment to account for PD delay (e.g. RISC-loaded siRNA kinetic for siRNA)	In general, an indirect response model can be applied to describe PK/PD relationship Additional mechanistic/empirical components may be required to capture further delay in tissue PK and/or PD responses, especially for conjugated oligonucleotides Humanized preclinical PD model is warranted to ensure PD transability Human PBPK/PD model is encouraged by incorporating available physiological and pharmacological parameters
Clinical applications		
FIH dose strategy	Human exposure limits using NOAEL (e.g. 1/10 NOAEL) Translational/mechanistic PK/PD or PBPK modeling to guide FIH dose range selection	Human exposure limits using NOAEL or a fraction of NOAEL depending on nonclinical toxicity findings and differences in clinical and nonclinical species Translational/mechanistic PK/PD or PBPK/PD modeling to better predict human efficacious dose and dosing frequency to inform FIH starting dose selection to ensure achievement of desirable maximum and sustained PD response to account for differences in patients if applicable
Clinical dose optimization	The typical M&S applications for other modalities are also applicable to oligonucleotides: SAD/MAD to Phase 2: establish clinical population PK/PD model based on emerging clinical data Refine dose escalation/selection in SAD/MAD studies Identify covariates affecting population PK/PD Inform Phase 2a/2b dose selection Phase 2 to Phase 3: update population PK/PD models using Phase 2 data, exposure-safety modeling, clinical trial simulations To inform Phase 3 dose selection	In addition to the typical clinical M&S practices, Clinical population PK/PD models for oligonucleotides may use translational PK/PD model as a prior as tissue exposure in humans is often unavailable. Human PBPK/PD model can be refined with emerging clinical data to increase confidence in prediction It is important to account for patient characteristics (e.g. baseline target levels) in clinical M&S Dose-response model may also be needed for clinical trial simulations for Phase 2b and Phase 3 study design Assuming dosing frequency established in Phase 1 and/or Phase 2a studies using population PK/PD approach
Clinical cardiac safety and special population studies	TQT waiver: Low risk of QT prolongation for oligonucleotides Inconsistency across programs/organizations in TQT waiver using concentration-QTc modeling Organ Impairment: Inconsistent M&S applications to support organ impairment study waiver/reduced design Pediatrics: Pediatric PK, dose and regimen prediction	TQT waiver: Concentration-QTc modeling of SAD and MAD data as a routine practice to support TQT waiver Organ Impairment: Routine population PK/PD modeling to evaluate impact of various degrees of organ impairment Verified PBPK/PD model to be leveraged to estimate impact of various degrees of organ impairment To support study waiver, reduced design or dose adjustment in populations with organ impairment Pediatrics: Population PK/PD model extrapolation, or pediatric PBPK/PD to predict pediatric dose and regimen
Regulatory applications		
Evaluation of intrinsic and extrinsic factors for the label	Quantitative evaluation of these factors is conducted primarily using a population PK analysis with increasing use of population PK/PD To inform the effects of intrinsic and extrinsic factors on clinical PK and PD in the label	Given the disconnect in plasma PK and PD for these modalities, robust population PK/PD modeling analysis is recommended using relevant data from all clinical studies and including intrinsic and extrinsic factors in covariate analysis To inform the effects of intrinsic and extrinsic factors on clinical PK and PD in the label
Confirmation of therapeutic dose and regimen for the label	Quantitative assessment of clinical efficacy and safety endpoints To support therapeutic dose in the label	Robust population PK/PD model, and/or exposure/dose-response model for efficacy and safety using relevant data from all clinical studies To support dose recommendation in the label with maximum benefit/risk ratio To support dose adjustment for significant intrinsic or extrinsic factors if warranted

**Table 7.** PK/PD modeling strategies used in translational and clinical development of approved GalNAc-conjugated siRNAs

PK/PD model type	Model description	Drugs approved	Knowledge gap
Hybrid animal PK/human PD model	Correlate observed animal liver concentration of siRNA, RISC-loaded siRNA level and changes in target mRNA activity to observed human PD data via allometric scaling	Givlaari™ (givosiran) Oxlumo™ (lumasiran)	The model can fit the central tendency of PD data reasonably with uncertainty in unmeasured human PK in the liver Note that Liver clearance for siRNA translated from preclinical models to humans need to be further optimized [70]
Kinetic-pharmacodynamic (K-PD) model	Correlate dose to human PD via a hypothetical liver effect compartment with biophase kinetics and turnover process of response of a drug	Leqvio™ (inclisiran)	The model adequately fit PD data with reasonable precision with no use of PK exposure data and a hypothetical liver effect compartment [71]
Indirect response PK/PD model	Utilize predicted individual blood concentrations from the population PK model to drive efficacy effect with a drug concentration effect compartment	Onpattro™ (patisiran) Rivfloza™ (nedosiran)	PD response at the target organ is linked to PK assumed as linear drug uptake into and linear elimination from the target organ. However, good model fit has been observed on longitudinal PD response [72, 73]

In general, the initial computational model for oligonucleotides can be established using nonclinical data for translational purposes (as outlined in Table 7) to guide FIH dose projections. The model can then be further refined when clinical data become available as a part of SAD and MAD studies to inform dose selection for phases 2 and 3 clinical studies, and in special populations such as organ-impaired (see the ‘Clinical pharmacology’ section), and ultimately support recommendation of therapeutic dose(s) in drug product label.

## Pharmacokinetic/pharmacodynamic modeling and simulation

### siRNA

Since siRNA was discovered and described by Fire *et al.*, in 1998, significant insights into its mechanisms and ADME properties have emerged [66]. Challenges persist in the translational space for siRNA therapies. Ayyar *et al.* developed a minimal PBPK/PD model for siRNA disposition and gene silencing [67]. This model included ASGPR-mediated liver uptake and showed some success in translating mouse *in vitro* data to *in vivo* activity, using allometry to scale parameters for larger species.

K-PD models estimate dose-response relationships using PD or biomarker data without relying on empirical concentration measurements. This approach is particularly relevant for siRNA therapeutics, which often exhibit short plasma half-lives relative to the duration of their PD effects. Additionally, direct measurement of target tissue concentrations is challenging, making K-PD modeling an attractive alternative [68]. However, Boianelli *et al.* analyzed biomarker data from mice, monkeys, and humans for 11 GalNAc-conjugated siRNAs and reported limited confidence in several estimated parameters [69]. While trends such as longer biophase half-life and potentially lower ED50 in humans were observed, many parameters lacked consistent cross-species patterns [68, 69].

These findings suggest that due to differences in targets and biological systems, K-PD parameter estimates may not be reliably scaled across species as is often done with small molecules. A promising alternative is the development of platform-based PK/PD models that account for both siRNA target biology and compound-specific clearance or biodistribution characteristics. If changes to the siRNA sequence do not alter the PK of the construct, platform K-PD models using fixed PK inputs could help predict target engagement across different constructs. Table 7 summarizes the strategies and considerations used in applying K-PD and platform PK/PD approaches for siRNA drug development.

### ASOs

Models for peripherally administered ASOs, siRNAs, and GalNAc-conjugated ASOs/siRNAs have shown similar structures [74]. Compartmental PK that considers tissue concentration as the site of action can effectively describe PK/PD behavior. Indirect-response models capture delayed PD responses due to downstream translation and turnover. Additional compartments may be needed to account for the delay in transcription and degradation of ASOs/siRNAs in tissues [74–76].

Research suggests that human PK for peripherally administered ASOs is highly translatable from NHPs [77, 78], while the translatability for intrathecally delivered ASOs is still pending further clinical validation [79]. PD translation may depend on the target and is more predictable from nonclinical species if the target biology is conserved or if humanized animal models are used in evaluation.

## Physiologically based pharmacokinetic modeling and simulation

### siRNA

Most approved siRNA therapeutics are GalNAc-siRNAs that target the liver after subcutaneous administration, aimed at treating rare diseases and increasingly in more prevalent indications. A minimal PBPK/PD model for GalNAc-siRNAs, developed by [67] for fitusiran and givosiran, was adapted into a broader platform framework and tested for inclisiran and vutrisiran [80]. Key parameters affecting translation success include hepatic endosomal degradation rates, fractional endosomal escape, RISC-loading, and degradation kinetics, and *in vivo* potency. This model-informed approach can help prioritize experiments for new GalNAc-siRNAs, optimizing translation strategies.

Additionally, pathophysiological models reflecting organ impairment have been developed [80] to assess how organ impairment mechanisms affect the PK, PD, and safety of GalNAc-siRNAs. While dedicated studies on organ impairment and GalNAc-siRNAs are limited—especially given their focus on rare diseases—data from inclisiran and vutrisiran in relevant patient populations were utilized to validate the model and its assumptions. These predictive tools can aid clinical pharmacology strategies to inform dosing considerations in organ impaired populations and guide decisions on whether dedicated clinical studies are necessary. Furthermore, such mechanistic modeling approaches allows for the identification of specific knowledge gaps to guide future research in this rapidly evolving field.

### Antisense oligonucleotides

Intrathecally administered ASOs are promising treatments for neurological diseases, such as nusinersen for spinal muscular atrophy (SMA) and tofersen for amyotrophic lateral sclerosis (ALS). A physiologically based pharmacokinetic (PBPK) model has been effective in characterizing ASO distribution across various organs and tissues, guiding FIH study designs [79]. After intrathecal injection, ASO concentrations can be measured in the CSF, spinal cord, liver, kidney, plasma, and specific brain regions like the cortex and hippocampus. This PK model can be linked to a PD model, which includes parameters like the half-maximal inhibitory concentration (IC50) and the turnover rate of the target.

By predicting target engagement, the model aids in determining the appropriate dose and sampling strategy for initial human studies. If the minimum recommended starting dose is too high, the PBPK model provides a more rational approach for selecting a safer dose that adequately captures the exposure-response relationship. While PBPK modeling can be validated with available human PK/PD data, such data are often lacking for brain tissues, limiting predictability. Conducting sensitivity analyses on ASO levels in target tissues is recommended to evaluate potential outcomes for target engagement.

To enhance the development efficiency of oligonucleotides, extending PBPK modeling to include target pharmacology and pathology (e.g. PBPK-PD or systems pharmacology approaches) could further improve model predictability as has been demonstrated for GalNAc-siRNAs [67, 80].

### Knowledge gaps, data limitations, and modeling challenges

Several knowledge gaps and data limitations were identified in industry-wide discussions making the development, validation, and application of computational approaches for these modalities challenging, including (but not limited to):

- Mechanisms of hepatic endosomal escape and RISC kinetics: For GalNAc-siRNAs, additional fundamental research is necessary to better understand the mechanism and kinetics of hepatic endosomal escape to model the free drug available for RISC-loading [81]. Similarly, understanding the kinetics of siRNA loading to RISC complex and subsequent degradation kinetics of RISC-loaded siRNA and its translation potential across species requires further investigation.
- Limited case studies: While hepatic clearance translation appears promising, it is based on limited data. More diverse studies on GalNAc-siRNAs are needed to strengthen confidence in current translation methods.
- Modeling approach-based limitations: For example, K-PD models do not require empirical tissue PK data but depend on biomarker and/or PD endpoints. While this saves tissue sampling burden (which is often not possible in human studies), the translatability of these biomarkers and target-engagement endpoints should be considered carefully when scaling from preclinical species to humans.
- Biomarker challenges: Circulating biomarkers are attractive to assess target engagement, however these data are distal to the site of target engagement for mRNA knockdown in the liver and often influenced by several physiological steps between target knockdown in liver and presentation of biomarker in the systemic circulation. These factors must be carefully considered when applying computational modeling approaches to translate preclinical data for FIH dose predictions.
- Data gaps: Modeling brain-targeted ASOs is challenging due to limited data and the lack of accessible human brain data for validation. Nonhuman primates are currently the most translatable preclinical species for predicting human brain and CSF PK. However, the long half-life of ASOs in the brain and the high cost of primate studies make it difficult to obtain comprehensive brain-based datasets. In clinical development, PK measurements and PD biomarkers from CSF are often used as brain samples are impractical to obtain. This creates additional challenges when the relationship between brain and CSF PK/PD is not well understood.

While we generally apply similar PKPD and PBPK strategies for siRNA and ASO, the actual modeling approach should be tailored to each specific case. Factors such as the target tissue (e.g. GalNAc-conjugated versus other conjugated or naked oligonucleotides), route of administration (e.g. systemic versus intrathecal), and the extent of existing knowledge or data gaps, as discussed above, can influence the selection of an optimal modeling strategy. As such, while computational tools hold significant promise for improving drug development efficiency, particularly for these modalities, they remain an area of active research. With increasing number of approved therapies, ongoing efforts are focused on leveraging these case studies to continue improving model acceptance standards comparable to other established therapeutic classes (small molecules and certain biologics) where such approaches have progressed beyond supplementary evidence to supporting regulatory and clinical waivers.

## Clinical pharmacology

### Characterization of QTc prolongation

Health authorities recommend evaluating the cardiac safety of Oligonucleotide therapeutics according to ICH E14 and S7B guidelines [82, 83], traditionally applied to small molecules. However, oligonucleotides have a larger molecular weight (6–20 kDa), which limits their interaction with hERG ion channels and results in low distribution volume due to their hydrophilic, negatively charged nature and low membrane permeability, leading to minimal exposure to cardiac myocytes [84]. Consequently, the risk of QTc prolongation with oligonucleotides is considered very low. For locally administered oligonucleotides like nusinersen and tofersen, plasma concentrations are significantly lower than those in CSF, further reducing cardiac safety risks [85–87] (https://www.accessdata.fda.gov/drugsatfda_docs/nda/2016/209531Orig1s000ClinPharmR.pdf). Studies have shown that marketed oligonucleotides tested in hERG assays did not exhibit inhibitory effects, and no QTc prolongation was observed in NHP studies [88].

To date, no clinically significant QT prolongation has been reported for approved oligonucleotides. Most Phase 1 trials included ECG monitoring for concentration-QTc response analysis, and dedicated QTc studies for mipomersen, inclisiran, and volanesorsen found no significant prolongation (Table 8) [77, 89].

**Table 8.** Summary of clinical ECG monitoring, dedicated dTQT, and organ impairment studies for approved oligonucleotide therapeutics

Compound (FDA/EMA approval)	Class	Modification/ bioconjugation	Adminis-tration route	Indication	Clinical ECG monitoring (Conc-QTc)	Dedicated TQT study	Hepatic impairment (HI) study	Renal impairment (RI) study
Mipomersen (2013)	ASO	2′MOE PS	SC	HoFH	Yes	Yes	NA	NA
Eteplirsen (2016)	ASO	PMO	IV	DMD	Yes	No	NA	NA
Nusinersen (2016)	ASO	2′MOE PS	IT	SMA	Yes	No	NA	NA
Patisiran (2018)	siRNA	LNP	IV	hATTR PN	Yes	No	NA (PopPK)	NA (PopPK)
Inotersen (2018)	ASO	2′MOE PS	SC	hATTR PN	Yes	No	NA (PopPK)	NA (PopPK)
Givosiran (2019)	siRNA	GalNAc	SC	Acute hepatic porphyria	Yes	No	NA (PopPK)	NA (PopPK)
Golodirsen (2019)	ASO	PMO	IV	DMD	Yes	Pending^b^	NA	Yes (Stage 2/3 CKD, *n* = 8 × 2 versus matched HV)
Volanesorsen (2019)*	ASO	2′MOE PS	SC	FCS	Yes	Yes	NA	NA
Viltolarsen (2020) ^1*^	ASO	PMO	IV	DMD	Yes	Pending^b^	NA	NA (∼75% renally cleared intact)
Lumasiran (2020)	siRNA	GalNAc	SC	PH1	Yes	No	NA (PopPK)	NA (PopPK)
Casimersen (2021)	ASO	PMO	IV	DMD	Yes	Pending^b^	NA (≥90% renally cleared intact)	Yes (Stage 2/3 CKD, *n* = 8 × 2 versus matched HV)
Inclisiran (2021)	siRNA	GalNAc	SC	ASCVD or HeFH	Yes	Yes	Yes (*n* = 10, 6 for mild and moderate HI versus *n* = 12 HV)	Yes (*n* = 8, 8, 7 for mild, moderate and severe RI versus *n* = 8 HV)
Vutrisiran (2022)	siRNA	GalNAc	SC	hATTR PN	Yes	No	NA (PopPK)	NA (PopPK)
Tofersen (2023)	ASO	2′MOE PS	IT	ALS	Yes	No	NA	NA
Nedosiran (2023)	siRNA	GalNAc	SC	PH1	Yes	No	PopPK	Yes
Eplontersen (2023)	ASO	2′MOE PS and GalNAc	SC	hATTR PN	Yes	No	NA (PopPK)	NA (PopPK)

a1 Only approved by EMA, not FDA.

b2 Pending: waiver of TQT study is not granted at the moment when this manuscript is written

ALS: amyotrophic lateral sclerosis; ASCVD: atherosclerotic cardiovascular disease; ASO: antisense oligonucleotide; DMD: Duchenne,muscular dystrophy; FCS: familial chylomicronemia syndrome; GalNAc: N-acetylgalactosamine; hATTR PN: hereditary transthyretin amyloidosis with polyneuropathy; HeFH: heterozygous familial hypercholesterolemia; HoFH: homozygous familial hypercholesterolemia; IT: intrathecal; IV: intravenous; LNP: lipid nanoparticle; 2′MOE: methoxyethyl; PS: phosphorothioate; PH1: primary hyperoxaluria type 1; PMO: phosphorodiamidate morpholino oligomer; SC: subcutaneous; siRNA: small interfering RNA; SMA: spinal muscular atrophy.

### IQ recommendations

- Toxicology studies: Conduct repeat-dose toxicology studies in relevant species alongside cardiovascular safety telemetry to assess QTc prolongation potential.
- Plasma concentration-QTc analysis: Use high-quality ECG recordings and time-matched PK sampling in early Phase studies to satisfy E14 guidance and potentially eliminate the need for additional QT measurements in later phases of clinical development.
- *In-vitro* hERG testing: hERG assays may be performed prior to FIH dosing to evaluate delayed repolarization risks.
- Engaging with the FDA’s Interdisciplinary Review Team for Cardiac Safety Studies is advised before the end of Phase 2 when predictions of the maximum therapeutic dose and high clinical exposure information may be available.

Given the low proarrhythmic potential indicated by existing data for this modality, a streamlined approach for evaluating QTc prolongation risks for oligonucleotides may be considered in the future.

### Evaluation of drug–drug interaction

The likelihood of oligonucleotides being affected by CYP modulators is low. Unlike small molecules, oligonucleotides are not substrates for cytochrome P450 (CYP450) enzymes or UDP-glucuronosyltransferases (UGT) [90]. They are hydrolyzed by endonucleases and exonucleases, meaning that CYP and UGT modulators are not expected to interact with oligonucleotides. The FDA acknowledges this lack of DDI (drug-drug interaction) potential in its guidance [52]. Oligonucleotides are also generally not substrates for major efflux transporters like P-glycoprotein (P-gp) and BCRP, or hepatic and renal uptake transporters [63]. Consequently, modulators of these transporters are not anticipated to significantly impact oligonucleotide PK. As perpetrators, oligonucleotides show low DDI potential with CYP enzymes or transporters. Comprehensive evaluations of 2′-MOE-ASOs and GalNAc-conjugated siRNAs found no significant interactions at clinically relevant concentrations [91]. Among the FDA-approved oligonucleotides, only givosiran exhibited some interactions due to its unique PD effect, specifically moderate inhibition of CYP1A2 and CYP2D6, with minor effects on CYP3A4 and CYP2C19 [36, 92, 93] (https://www.accessdata.fda.gov/drugsatfda_docs/nda/2019/212194Orig1s000TOC.cfm).

### IQ recommendations for DDI

The FDA recommends assessing oligonucleotides as potential inhibitors or inducers of CYP enzymes or transporters *in vitro*, guiding sponsors to refer to the relevant FDA DDI guidance [94]. Current best practice allows using *in vitro* DDI studies to justify waivers for clinical DDI studies. There have been no reported oligonucleotide DDIs in regulatory filings, apart from the mechanism-based DDI observed with givosiran. The IQ Consortium Nucleic Acid Clinical Pharmacology Working Group believes that standard preclinical evaluations for oligonucleotide interactions with CYP enzymes or transporters are unnecessary. Sponsors should align their strategies with health authorities, particularly in pre-IND meetings. Clinical DDI studies may be warranted if mechanism-based or disease-drug interactions are anticipated with oligonucleotide administration [95].

### Impact of organ impairment on oligonucleotide therapeutics

**Figure 2.**
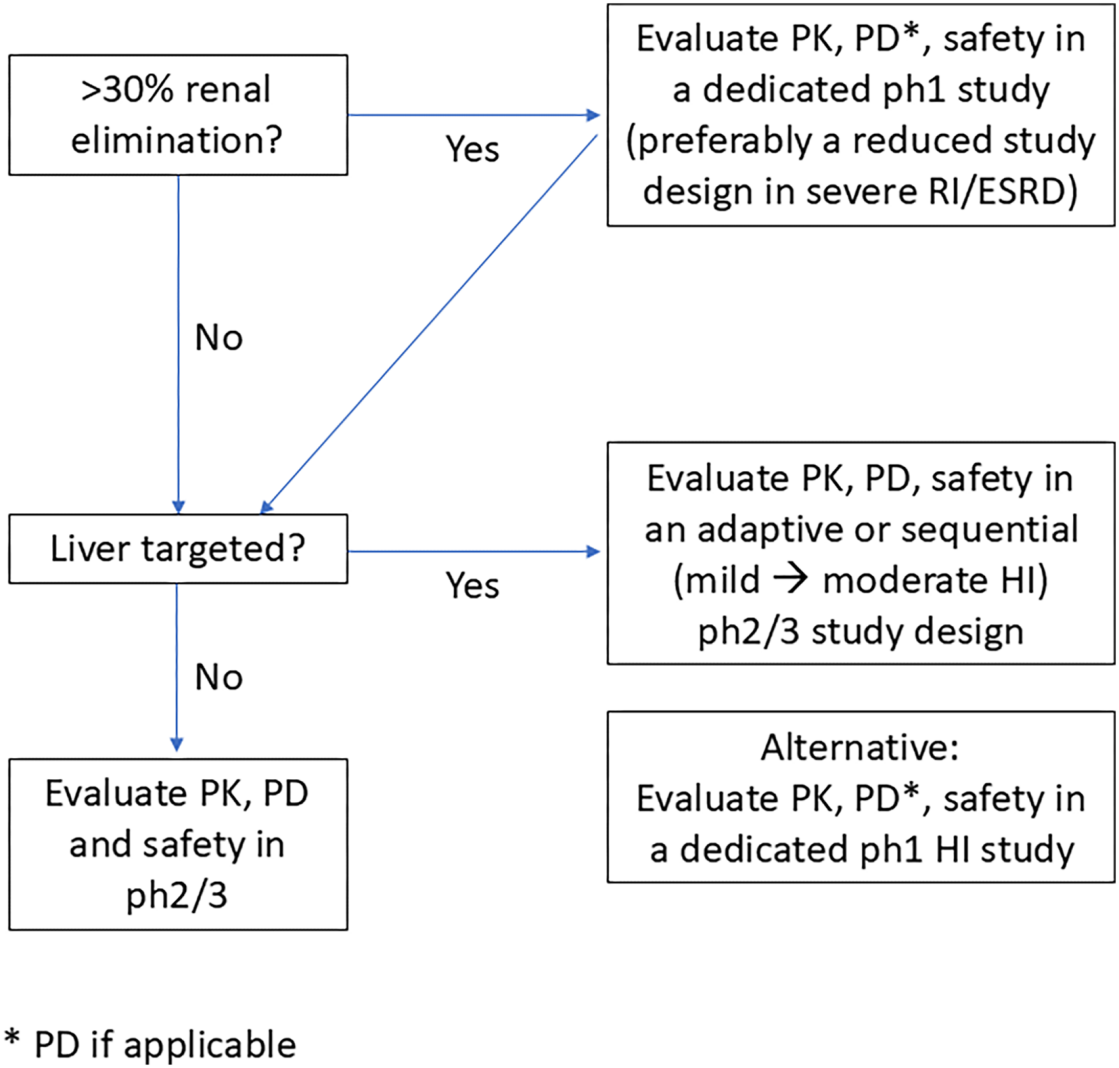
Organ impairment study decision tree.

Systemically administered oligonucleotides primarily accumulate in the liver and are excreted by the kidneys, making it essential to consider HI and RI in clinical development (Table 8) [24, 95–97]. Figure 2 illustrates a decision tree to guide organ impairment clinical studies with details provided below. Preclinical studies should inform the roles of the liver and kidneys in oligonucleotide elimination, and mechanistic PBPK modeling can estimate the effects of organ impairment [80].

### Renal impairment (RI)

Some oligonucleotides, like givosiran, have shown increases in serum creatinine and decreased eGFR, necessitating monitoring of renal safety biomarkers. If an oligonucleotide is minimally renally excreted (<30%), enrolling subjects with varying degrees of RI in late-phase trials is advisable, provided safety data supports it. If an oligonucleotide is significantly renally excreted (>30%) or safety data is insufficient, a dedicated RI study may be conducted, typically in parallel with phase 3 trials after consultation with health authorities. The effect of RI on systemic exposure can also be assessed using quantitative modeling.

General design considerations for dedicated RI studies apply to oligonucleotides. A reduced study design can be considered when conducting dedicated RI studies.

Overall, oligonucleotides are unlikely to show clinically meaningful PK, PD, or safety impacts in RI patients (Table 8). Even if systemic exposure changes are observed, dose adjustments may not be necessary. For example, changes in exposure for golodirsen and casimersen in CKD patients did not lead to dose adjustments for DMD patients [98, 99] (https://www.accessdata.fda.gov/drugsatfda_docs/label/2021/213026lbl.pdf), (https://www.accessdata.fda.gov/drugsatfda_docs/label/2021/211970s002lbl.pdf). Similarly, inclisiran showed exposure changes in RI studies, but no dose adjustments were recommended due to consistent PD and safety outcomes [100].

### Hepatic impairment

According to FDA guidelines [52], including HI patients in clinical trials for nonliver-targeted oligonucleotides is encouraged. The effect of HI on PK and PD can be assessed using quantitative modeling (Table 8). For liver-targeted oligonucleotides, an adaptive or sequential design may assess PK, safety, and efficacy in patients with varying HI levels. If this is not unfeasible, a dedicated phase 1 HI study may be needed. While HI can lead to increased plasma concentrations for liver-targeted oligonucleotides, this does not necessarily imply a need for dose adjustment [101, 102]. For instance, inclisiran showed less pronounced LDL-C reduction in moderate HI patients but did not require dose adjustments [89]. GalNAc-siRNAs are often designed to saturate the ASGPR receptor, limiting the benefits of increased dosing.

### Ethnopharmacology evaluation

Limited data is available on the effect of race/ethnicity on oligonucleotides PK but differences may exist based on target receptor expression. It is recommended to include a diverse population in phase 2/3 clinical studies from which covariate population PK analysis can be used to examine the effect of race. Covariate popPK analysis of the ASOs bepirovirsen, danvatirsen and inotersen, GalNAc-ASO GSK3389404, and the LNP-siRNA patisiran showed no significant effect of race on PK [103–107]. A phase 1 bridging PK study was done for the GalNAc-siRNA JNJ-73763989 in Chinese and Japanese healthy participants showing no difference in PK [108].

## Conclusion

The development of siRNA and ASO therapies requires consideration of multiple aspects. These include bioconjugation (linking the drug to molecules that guide it to the right cells), bioanalysis (measuring the drug in tissues or blood), biotransformation (how the drug is broken down in the body), tissue distribution, computational modeling, and clinical pharmacology. While regulatory guidelines have started to emerge, they still lack detailed and consistent direction for many of these aspects.

This manuscript, developed by the IQ Consortium Nucleic Acid Working Group, brings together insights from industry experts working on these therapies. It highlights critical gaps in current guidance and provides practical recommendations, especially in the areas of bioanalytical methods, biotransformation assessment, and tissue distribution studies. One major gap is the lack of clear guidance on how to evaluate bioconjugates—modified forms of the drugs designed to improve delivery.

The group also stresses the growing importance of computational M&S in drug development. These tools can help predict how a drug behaves in the body, support dose selection, and improve understanding of how tissue-specific drug levels (rather than plasma levels) drive the therapeutic response. This is particularly relevant for siRNAs and ASOs, which act inside cells in specific tissues.

Finally, a recent draft guidance from the FDA on clinical pharmacology for oligonucleotide therapeutics has been reviewed. While it is a positive step, the group offers further recommendations to strengthen regulatory clarity and consistency. Continued collaboration between industry and regulatory agencies will be essential to support the safe and effective development of these innovative treatments, especially for patients with special needs.

## Data Availability

No new data were generated or analyzed in support of this research.

## Notes

Present address: Translational Immunology, Vividion Therapeutics, San Diego, CA 92121, United States

## References

[1] Biessen EAL, Van Berkel TJC N-acetyl galactosamine targeting: paving the way for clinical application of nucleotide medicines in cardiovascular diseases. Arterioscler Thromb Vasc Biol. 2021; 41:2855–65. 10.1161/ATVBAHA.121.316290. 34645280

[2] Mullard A Antibody-oligonucleotide conjugates enter the clinic. Nat Rev Drug Discov. 2022; 21:6–8. 10.1038/d41573-021-00213-5. 34903879

[3] Patil NA Conjugation approaches for peptide-mediated delivery of oligonucleotides therapeutics. Aust J Chem. 2021; 75:24–33. 10.1071/CH21131.

[4] Hoang Thi TT, Pilkington EH, Nguyen DH et al. The importance of poly(ethylene glycol) alternatives for overcoming PEG immunogenicity in drug delivery and bioconjugation. Polymers. 2020; 12:298–319. 10.3390/polym12020298. 32024289 PMC7077443

[5] Steurer M, Montillo M, Scarfo L et al. Olaptesed pegol (NOX-A12) with bendamustine and rituximab: a phase IIa study in patients with relapsed/refractory chronic lymphocytic leukemia. Haematologica. 2019; 104:2053–60. 10.3324/haematol.2018.205930. 31097627 PMC6886437

[6] Shokrzadeh N, Winkler AM, Dirin M et al. Oligonucleotides conjugated with short chemically defined polyethylene glycol chains are efficient antisense agents. Bioorg Med Chem Lett. 2014; 24:5758–61. 10.1016/j.bmcl.2014.10.045. 25453815 PMC4263527

[7] Tran P, Weldemichael T, Liu Z et al. Delivery of oligonucleotides: efficiency with lipid conjugation and clinical outcome. Pharmaceutics. 2022; 14:342 10.3390/pharmaceutics14020342. 35214074 PMC8879684

[8] Roberts TC, Langer R, Wood MJA Advances in oligonucleotide drug delivery. Nat Rev Drug Discov. 2020; 19:673–94. 10.1038/s41573-020-0075-7. 32782413 PMC7419031

[9] Sutton JM, Guimaraes GJ, Annavarapu V et al. Current State of oligonucleotide characterization using liquid chromatography-mass spectrometry: insight into critical issues. J Am Soc Mass Spectrom. 2020; 31:1775–82. 10.1021/jasms.0c00179. 32812756

[10] Gawlig C, Hanci G, Ruhl M Quantification of oligonucleotides using tandem mass spectrometry with isobaric internal standards. Int J Mol Sci. 2023; 24:14691–702. 10.3390/ijms241914691. 37834137 PMC10573027

[11] Sips L, Ediage EN, Ingelse B et al. LC-MS quantification of oligonucleotides in biological matrices with SPE or hybridization extraction. Bioanalysis. 2019; 11:1941–54. 10.4155/bio-2019-0117. 31829054

[12] Yuan L, Dupuis JF, Mekhssian K A novel hybridization LC-MS/MS methodology for quantification of siRNA in plasma, CSF and tissue samples. Molecules. 2023; 28:1618–36. 36838605 10.3390/molecules28041618 PMC9967190

[13] Li J, Liu J, Enders J et al. Discovery of a novel deaminated metabolite of a single-stranded oligonucleotide *in vivo* by mass spectrometry. Bioanalysis. 2019; 11:1955–65. 10.4155/bio-2019-0118. 31829055

[14] Agrawal K, Kang L, Ji S et al. Evaluating the use of locked nucleic acid capture probes in hybrid LC-MS/MS analysis of siRNA analytes. Bioanalysis. 2023; 15:1129–46. 10.4155/bio-2023-0079. 37638814

[15] Agrawal K, Calliste LK, Ji S et al. Comparison of multiple bioanalytical assay platforms for the quantitation of siRNA therapeutics. Bioanalysis. 2024; 16:651–67. 10.1080/17576180.2024.2350266. 39254503 PMC11389733

[16] Kim J, El Zahar NM, Bartlett MG In vitro metabolism of 2′-ribose unmodified and modified phosphorothioate oligonucleotide therapeutics using liquid chromatography mass spectrometry. Biomed Chromatogr. 2020; 34:e4839 10.1002/bmc.4839. 32246854

[17] Stulz R, Milligan F, Stovold C et al. ^34^S-SIL of PCSK9-active oligonucleotide as tools for accurate quantification by mass spectrometry. Nucleic Acid Ther. 2021; 31:375–81. 10.1089/nat.2020.0915. 33978476

[18] Li P, Gong Y, Kim J et al. Hybridization liquid chromatography-tandem mass spectrometry: an alternative bioanalytical method for antisense oligonucleotide quantitation in plasma and tissue samples. Anal Chem. 2020; 92:10548–59. 10.1021/acs.analchem.0c01382. 32628461

[19] Thayer MB, Lade JM, Doherty D et al. Application of locked nucleic acid oligonucleotides for siRNA preclinical bioanalytics. Sci Rep. 2019; 9:3566 10.1038/s41598-019-40187-4. 30837588 PMC6401054

[20] Kotapati S, Deshpande M, Jashnani A et al. The role of ligand-binding assay and LC-MS in the bioanalysis of complex protein and oligonucleotide therapeutics. Bioanalysis. 2021; 13:931–54. 10.4155/bio-2021-0009. 33998268

[21] Castellanos-Rizaldos E, Brown CR, Dennin S et al. RT-qPCR methods to support pharmacokinetics and drug mechanism of action to advance development of RNAi therapeutics. Nucleic Acid Ther. 2020; 30:133–42. 10.1089/nat.2019.0840. 32202961

[22] Pei Y, Hancock PJ, Zhang H et al. Quantitative evaluation of siRNA delivery *in vivo*. RNA. 2010; 16:2553–63. 10.1261/rna.2255810. 20940339 PMC2995415

[23] Nair JK, Attarwala H, Sehgal A et al. Impact of enhanced metabolic stability on pharmacokinetics and pharmacodynamics of GalNAc-siRNA conjugates. Nucleic Acids Res. 2017; 45:10969–77. 10.1093/nar/gkx818. 28981809 PMC5737438

[24] McDougall R, Ramsden D, Agarwal S et al. The nonclinical disposition and pharmacokinetic/pharmacodynamic properties of N-acetylgalactosamine-conjugated small interfering RNA are highly predictable and build confidence in translation to human. Drug Metab Dispos. 2022; 50:781–97. 10.1124/dmd.121.000428. 34154993

[25] Turski MK, Albertolle ME Utilizing droplet digital polymerase chain reaction for siRNA quantitation in rodent plasma and tissue via stem-loop reverse transcription. Bioanalysis. 2024; 16:375–88. 10.4155/bio-2023-0228. 38380639

[26] Murphy A, Hill R, Berna M Bioanalytical approaches to support the development of antibody-oligonucleotide conjugate (AOC) therapeutic proteins. Xenobiotica. 2024; 54:552–562. 38607350 10.1080/00498254.2024.2339983

[27] Sela T, Manso M, Siegel M et al. Diligent design enables antibody-ASO conjugates with optimal pharmacokinetic properties. Bioconjug Chem. 2023; 34:2096–111. 10.1021/acs.bioconjchem.3c00393. 37916986

[28] Liu J, Li J, Tran C et al. Oligonucleotide quantification and metabolite profiling by high-resolution and accurate mass spectrometry. Bioanalysis. 2019; 11:1967–80. 10.4155/bio-2019-0137. 31829056

[29] Ji Y, Liu Y, Xia W et al. Importance of probe design for bioanalysis of oligonucleotides using hybridization-based LC-fluorescence assays. Bioanalysis. 2019; 11:1917–25. 10.4155/bio-2019-0154. 31637930

[30] Thayer MB, Humphreys SC, Chung KS et al. POE immunoassay: plate-based oligonucleotide electro-chemiluminescent immunoassay for the quantification of nucleic acids in biological matrices. Sci Rep. 2020; 10:10425 10.1038/s41598-020-66829-6. 32591626 PMC7319975

[31] Wang L, Ji C Advances in quantitative bioanalysis of oligonucleotide biomarkers and therapeutics. Bioanalysis. 2016; 8:143–55. 10.4155/bio.15.234. 26652713

[32] Li XQ, Elebring M, Dahlen A et al. *viv**o* metabolite profiles of an N-acetylgalactosamine-conjugated antisense oligonucleotide AZD8233 using liquid chromatography high-resolution mass spectrometry: a cross-species comparison in animals and humans. Drug Metab Dispos. 2023; 51:1350–61. 10.1124/dmd.123.001370. 37429729

[33] Weidolf L, Bjorkbom A, Dahlen A et al. Distribution and biotransformation of therapeutic antisense oligonucleotides and conjugates. Drug Discovery Today. 2021; 26:2244–58. 10.1016/j.drudis.2021.04.002. 33862193

[34] Beverly M, Hartsough K, Machemer L Liquid chromatography/electrospray mass spectrometric analysis of metabolites from an inhibitory RNA duplex. Rapid Comm Mass Spectrometry. 2005; 19:1675–82. 10.1002/rcm.1972. 15912467

[35] Shemesh CS, Yu RZ, Gaus HJ et al. Elucidation of the biotransformation pathways of a Galnac3-conjugated antisense oligonucleotide in rats and monkeys. Mol Ther Nucleic Acids. 2016; 5:e319 10.1038/mtna.2016.31. 27164023 PMC5014515

[36] USFDA GIVLAARI (givosiran)injection. 2019; (10 July 2025, date last accessed) https://www.accessdata.fda.gov/drugsatfda_docs/nda/2019/212194Orig1s000TOC.cfm.

[37] USFDA Nda-214103, Oxlumo. 2020; (10 July 2025, date last accessed) https://www.accessdata.fda.gov/drugsatfda_docs/nda/2020/214103Orig1s000TOC.cfm.

[38] Christensen JK, Bjornsdottir I, Sonesson A et al. Meeting report: DMDG peptide and oligonucleotide ADME workshop 2022. Xenobiotica. 2023; 53:332–7. 10.1080/00498254.2023.2223666. 37309582

[39] Rentel C, DaCosta J, Roussis S et al. Determination of oligonucleotide deamination by high resolution mass spectrometry. J Pharm Biomed Anal. 2019; 173:56–61. 10.1016/j.jpba.2019.05.012. 31121454

[40] Marlowe JL, Akopian V, Karmali P et al. Recommendations of the Oligonucleotide Safety Working Group’s Formulated Oligonucleotide Subcommittee for the safety assessment of formulated oligonucleotide-based therapeutics. Nucleic Acid Ther. 2017; 27:183–96. 10.1089/nat.2017.0671. 28609186

[41] McKenzie R, Fried MW, Sallie R et al. Hepatic failure and lactic acidosis due to fialuridine (FIAU), an investigational nucleoside analogue for chronic hepatitis B. N Engl J Med. 1995; 333:1099–105. 10.1056/NEJM199510263331702. 7565947

[42] Janas MM, Zlatev I, Liu J et al. Safety evaluation of 2′-deoxy-2′-fluoro nucleotides in GalNAc-siRNA conjugates. Nucleic Acids Res. 2019; 47:3306–20. 10.1093/nar/gkz140. 30820542 PMC6468299

[43] Lozac’h F, Christensen J, Faller T et al. ADME studies of [5-(3)H]-2′-O-methyluridine nucleoside in mice: a building block in siRNA therapeutics. Pharmacol Res Perspec. 2016; 4:e00209 10.1002/prp2.209. PMC4777266 26977299

[44] Tomassini N, D’Andrea A, Martino G et al. Zn(S,Se)-based superlattices and quantum wells: band offsets, excitons, linear and nonlinear optical properties. Phys Rev B. 1995; 52:11113–9. 10.1103/PhysRevB.52.11113. 9980210

[45] USFDA Nda-215515, Amvuttra. 2022; (10 July 2025, date last accessed) https://www.accessdata.fda.gov/drugsatfda_docs/nda/2022/215515Orig1s000TOC.cfm.

[46] EMA Inclisiran. 2019; (10 July 2025, date last accessed) https://www.ema.europa.eu/en/documents/assessment-report/leqvio-epar-public-assessment-report_en.pdf.

[47] USFDA NDA 213026 AMONDYS45 : drug approval package. 2021; (10 July 2025, date last accessed) https://www.accessdata.fda.gov/drugsatfda_docs/nda/2021/213026Orig1s000TOC.cfm.

[48] Basiri B, Xie F, Wu B et al. Introducing an In vitro liver stability assay capable of predicting the *in vivo* pharmacodynamic efficacy of siRNAs for IVIVC. Mol Ther Nucleic Acids. 2020; 21:725–36. 10.1016/j.omtn.2020.07.012. 32771924 PMC7415771

[49] PMDA Guideline for preclinical safety assessment of oligonucleotide therapeutics. 2020; (10 July 2025, date last accessed) https://www.pmda.go.jp/files/000236289.pdf.

[50] ICH M3(R2) Guidance on nonclinical Safety Studies for the conduct of human clinical trials and marketing authorization for pharmaceuticals. 2009; (10 July 2025, date last accessed) https://www.ema.europa.eu/en/documents/scientific-guideline/ich-guideline-m3r2-non-clinical-safety-studies-conduct-human-clinical-trials-and-marketing-authorisation-pharmaceuticals-step-5_en.pdf. 20349552

[51] USFDA Clinical pharmacology considerations for human radiolabeled mass balance studies. 2024; (10 July 2025, date last accessed) https://www.fda.gov/regulatory-information/search-fda-guidance-documents/clinical-pharmacology-considerations-human-radiolabeled-mass-balance-studies.

[52] USFDA Clinical pharmacology considerations for the development of oligonucleotide therapeutics: guidance for industry. 2024; (10 July 2025, date last accessed) https://www.fda.gov/regulatory-information/search-fda-guidance-documents/clinical-pharmacology-considerations-development-oligonucleotide-therapeutics.

[53] Li W, Vazvaei-Smith F, Dear G et al. Metabolite bioanalysis in drug development: recommendations from the IQ Consortium Metabolite Bioanalysis Working Group. Clin Pharma Ther. 2024; 115:939–53. 10.1002/cpt.3144. 38073140

[54] USFDA Safety testing of drug metabolites: guidance for industry. 2020; (10 July 2025, date last accessed) https://www.fda.gov/media/72279/download.

[55] ICH S6(R1) Preclinical safety evaluation of biotechnology-derived pharmaceuticals. 2011; (10 July 2025, date last accessed) https://www.database.ich.org/sites/default/files/S6_R1_Guideline_0.pdf. 10.1038/nrd822 12119749

[56] USFDA Nonclinical safety assessment of oligonucleotide-based therapeutics, guidance for industry. 2024; (10 July 2025, date last accessed) https://www.fda.gov/media/183496/download.

[57] Solon EG Autoradiography techniques and quantification of drug distribution. Cell Tissue Res. 2015; 360:87–107. 10.1007/s00441-014-2093-4. 25604842

[58] Viel T, Boisgard R, Kuhnast B et al. Molecular imaging study on *in vivo* distribution and pharmacokinetics of modified small interfering RNAs (siRNAs). Oligonucleotides. 2008; 18:201–12. 10.1089/oli.2008.0133. 18729822

[59] Mazur C, Powers B, Zasadny K et al. Brain pharmacology of intrathecal antisense oligonucleotides revealed through multimodal imaging. JCI Insight. 2019; e129240 4 10.1172/jci.insight.129240. 31619586 PMC6824309

[60] USFDA NDA-20961. Vitravene (fomivirsen). Pharmacology and Toxicology Review. 1998; (10 July 2025, date last accessed) https://www.accessdata.fda.gov/drugsatfda_docs/nda/98/20961_Vitravene_pharmr.pdf.

[61] USFDA Vitravene (Fomivirsen Sodium Intravitreal Injectable) injection. 1998; 20–961.(10 July 2025, date last accessed) https://www.accessdata.fda.gov/drugsatfda_docs/nda/98/20961_Vitravene.cfm.

[62] USFDA Nda-215887. QALSODY (tofersen). 2023; (10 July 2025, date last accessed) https://www.accessdata.fda.gov/drugsatfda_docs/nda/2023/215887Orig1s000OEList.pdf.

[63] Berman CL, Antonsson M, Batkai S et al. OSWG recommended approaches to the nonclinical pharmacokinetic (ADME) characterization of therapeutic oligonucleotides. Nucleic Acid Ther. 2023; 33:287–305. 10.1089/nat.2023.0011. 37590469 PMC10561745

[64] USFDA Development and licensure of vaccines to prevent COVID-19. 2023; (10 July 2025, date last accessed) https://www.fda.gov/regulatory-information/search-fda-guidance-documents/development-and-licensure-vaccines-prevent-covid-19.

[65] USFDA Platform technology designation program for drug development: guidance for industry. 2024; (10 July 2025, date last accessed) https://www.fda.gov/media/178938/download.

[66] Ranasinghe P, Addison ML, Webb DJ Small interfering RNA therapeutics in hypertension: a viewpoint on vasopressor and vasopressor-sparing strategies for counteracting blood pressure lowering by angiotensinogen-targeting small interfering RNA. J Am Heart Assoc. 2022; 11:e027694 10.1161/JAHA.122.027694. 36216481 PMC9673665

[67] Ayyar VS, Song D, Zheng S et al. Minimal physiologically based pharmacokinetic–pharmacodynamic (mPBPK-PD) model of N-acetylgalactosamine-conjugated small interfering RNA disposition and gene silencing in preclinical species and humans. J Pharmacol Exp Ther. 2021; 379:134–46. 10.1124/jpet.121.000805. 34413198

[68] Jacqmin P, Snoeck E, van Schaick EA et al. Modelling response time profiles in the absence of drug concentrations: definition and performance evaluation of the K-PD model. J Pharmacokinet Pharmacodyn. 2007; 34:57–85. 10.1007/s10928-006-9035-z. 17051439

[69] Boianelli A, Aoki Y, Ivanov M et al. Cross-species translation of biophase half-life and potency of GalNAc-conjugated siRNAs. Nucleic Acid Ther. 2022; 32:507–12. 10.1089/nat.2022.0010. 35867041 PMC9784597

[70] Lee J, Melch M, Robbie GJ Pharmacokinetic–pharmacodynamic model of urinary delta-aminolevulinic acid reduction after givosiran treatment in patients with acute hepatic porphyria. CPT Pharmacometrics Syst Pharmacol. 2023; 12:842–52. 10.1002/psp4.12957. 36883675 PMC10272304

[71] Gosselin NH, Schuck VJA, Barriere O et al. Translational population-pharmacodynamic modeling of a novel long-acting siRNA therapy, Inclisiran, for the treatment of hypercholesterolemia. Clin Pharma Ther. 2023; 113:328–38. 10.1002/cpt.2774. 36281788

[72] Goel V, Gosselin NH, Jomphe C et al. Population pharmacokinetic–pharmacodynamic model of serum transthyretin following Patisiran administration. Nucleic Acid Ther. 2020; 30:143–52. 10.1089/nat.2019.0841. 32175804

[73] Zhang S, Amrite A, Tan B et al. Nedosiran population pharmacokinetic and pharmacodynamic modelling and simulation to guide clinical development and dose selection in patients with primary hyperoxaluria type 1. Brit J Clin Pharma. 2024; 90:3176–89. 10.1111/bcp.16194. PMC11602945 39113219

[74] Gao X, Diep JK, Norris DA et al. Predicting the pharmacokinetics and pharmacodynamics of antisense oligonucleotides: an overview of various approaches and opportunities for PBPK/PD modelling. Expert Opin Drug Metab Toxicol. 2023; 19:979–90. 10.1080/17425255.2023.2283524. 37970635

[75] Yu RZ, Gunawan R, Post N et al. Disposition and pharmacokinetics of a GalNAc3-conjugated antisense oligonucleotide targeting human lipoprotein (a) in monkeys. Nucleic Acid Ther. 2016; 26:372–80. 10.1089/nat.2016.0623. 27500733

[76] Ahn JE, Terra SG, Liu J A population pharmacokinetic and pharmacokinetic–pharmacodynamic analysis of vupanorsen from phase I and phase II studies. CPT Pharmacometrics Syst Pharmacol. 2023; 12:988–1000. 10.1002/psp4.12969. 37170423 PMC10349191

[77] Yu RZ, Grundy JS, Henry SP et al. Predictive dose-based estimation of systemic exposure multiples in mouse and monkey relative to human for antisense oligonucleotides with 2′-o-(2-methoxyethyl) modifications. Mol Ther Nucleic Acids. 2015; 4:e218 10.1038/mtna.2014.69. 25602582 PMC4345302

[78] Jiang R, Hooshfar S, Rebecca Eno M et al. Factors influencing ADME properties of therapeutic antisense oligonucleotides: physicochemical characteristics and beyond. Curr Drug Metab. 2023; 24:536–52. 10.2174/1389200224666230418092626. 37076460

[79] Monine M, Norris D, Wang Y et al. A physiologically-based pharmacokinetic model to describe antisense oligonucleotide distribution after intrathecal administration. J Pharmacokinet Pharmacodyn. 2021; 48:639–54. 10.1007/s10928-021-09761-0. 33991294

[80] Lumen A, Zhang X, Dutta S et al. Predicting clinical pharmacokinetics/pharmacodynamics and impact of organ impairment on siRNA-based therapeutics using a mechanistic physiologically-based pharmacokinetic–pharmacodynamic model. Clin Pharma Ther. 2024; 115:1054–64. 10.1002/cpt.3160. 38282246

[81] Dowdy SF, Setten RL, Cui XS et al. Delivery of RNA therapeutics: the great endosomal escape!. Nucleic Acid Ther. 2022; 32:361–8. 10.1089/nat.2022.0004. 35612432 PMC9595607

[82] USFDA E14 Clinical evaluation of QT/QTc interval prolongation and proarrhythmic potential for non-antiarrhythmic drugs. 2005; (10 July 2025, date last accessed) https://www.fda.gov/media/71372/download. 16237860

[83] USFDA S7B Nonclinical evaluation of the potential for delayed ventricular repolarization (QT Interval Prolongation) by human pharmaceuticals. 2005; (10 July 2025, date last accessed) https://www.fda.gov/regulatory-information/search-fda-guidance-documents/s7b-nonclinical-evaluation-potential-delayed-ventricular-repolarization-qt-interval-prolongation. 16237859

[84] Yu RZ, Gunawan R, Geary RS et al. Lack of QT prolongation for 2′-O-methoxyethyl-modified antisense oligonucleotides based on retrospective exposure/response analysis of ten phase 1 dose-escalation placebo-controlled studies in healthy subjects. Nucleic Acid Ther. 2017; 27:285–94. 10.1089/nat.2017.0676. 28799823 PMC5649121

[85] Geary RS, Norris D, Yu R et al. Pharmacokinetics, biodistribution and cell uptake of antisense oligonucleotides. Adv Drug Deliv Rev. 2015; 87:46–51. 10.1016/j.addr.2015.01.008. 25666165

[86] Biliouris K, Gaitonde P, Yin W et al. A semi-mechanistic population pharmacokinetic model of Nusinersen: an antisense oligonucleotide for the treatment of spinal muscular atrophy. CPT Pharmacometrics Syst Pharmacol. 2018; 7:581–92. 10.1002/psp4.12323. 30043511 PMC6157691

[87] USFDA Nusinersen (SpinrazaTM), Clinical pharmacology and biopharmaceutics reviews. 2016; (10 July 2025, date last accessed) https://www.accessdata.fda.gov/drugsatfda_docs/nda/2016/209531Orig1s000ClinPharmR.pdf.

[88] Qu Y, Kirby R, Davies R et al. Time is a critical factor when evaluating oligonucleotide therapeutics in hERG assays. Nucleic Acid Ther. 2023; 33:132–40. 10.1089/nat.2022.0043. 36576986 PMC10066779

[89] Kallend D, Stoekenbroek R, He Y et al. Pharmacokinetics and pharmacodynamics of inclisiran, a small interfering RNA therapy, in patients with hepatic impairment. J Clin Lipidol. 2022; 16:208–19. 10.1016/j.jacl.2022.01.001. 35168913

[90] Geary RS Antisense oligonucleotide pharmacokinetics and metabolism. Expert Opin Drug Metab Toxicol. 2009; 5:381–91. 10.1517/17425250902877680. 19379126

[91] Shemesh CS, Yu RZ, Warren MS et al. Assessment of the drug interaction potential of unconjugated and GalNAc(3)-conjugated 2′-MOE-ASOs. Mol Ther Nucleic Acids. 2017; 9:34–47. 10.1016/j.omtn.2017.08.012. 29246313 PMC5602538

[92] Humphreys SC, Davis JA, Iqbal S et al. Considerations and recommendations for assessment of plasma protein binding and drug–drug interactions for siRNA therapeutics. Nucleic Acids Res. 2022; 50:6020–37. 10.1093/nar/gkac456. 35687098 PMC9226521

[93] Vassiliou D, Sardh E, Harper P et al. A drug–drug interaction study evaluating the effect of Givosiran, a small interfering ribonucleic acid, on cytochrome P450 activity in the liver. Clin Pharma Ther. 2021; 110:1250–60. 10.1002/cpt.2419. 34510420

[94] USFDA *In vitro* drug interaction studies: cytochrome *P*-450 enzyme- and transporter-mediated drug interactions. 2020; (10 July 2025, date last accessed) https://collections.nlm.nih.gov/catalog/nlm:nlmuid-101767646-pdf?_gl=1*lpmax4*_ga*OTYxOTE3ODEyLjE2NzI4NjA0NDA.*_ga_7147EPK006*MTczMTQ2Njc4NC4xLjAuMTczMTQ2Njc5MS4wLjAuMA..*_ga_P1FPTH9PL4*MTczMTQ2Njc4NC4xLjAuMTczMTQ2Njc5MS4wLjAuMA.

[95] Jing X, Arya V, Reynolds KS et al. Clinical pharmacology of RNA interference-based therapeutics: a summary based on Food and Drug Administration-approved small interfering RNAs. Drug Metab Dispos. 2023; 51:193–8. 10.1124/dmd.122.001107. 36332914 PMC9900864

[96] Migliorati JM, Jin J, Zhong XB siRNA drug Leqvio (inclisiran) to lower cholesterol. Trends Pharmacol Sci. 2022; 43:455–6. 10.1016/j.tips.2022.02.003. 35307191 PMC9802187

[97] Abosalha AK, Boyajian J, Ahmad W et al. Clinical pharmacology of siRNA therapeutics: current status and future prospects. Expert Rev Clin Pharmacol. 2022; 15:1327–41. 10.1080/17512433.2022.2136166. 36251525

[98] USFDA AMONDYS 45 (casimersen) injection. 2021; (10 July 2025, date last accessed) https://www.accessdata.fda.gov/drugsatfda_docs/label/2021/213026lbl.pdf.

[99] USFDA VYONDYS 53 (golodirsen) injection. 2019; (10 July 2025, date last accessed) https://www.accessdata.fda.gov/drugsatfda_docs/label/2021/211970s002lbl.pdf.

[100] Wright RS, Collins MG, Stoekenbroek RM et al. Effects of renal impairment on the pharmacokinetics, efficacy, and safety of Inclisiran: an analysis of the ORION-7 and ORION-1 studies. Mayo Clin Proc. 2020; 95:77–89. 10.1016/j.mayocp.2019.08.021. 31630870

[101] Kakuda M, Morais N, Kwoen J et al. Enhanced temperature stability of threshold current of InAs/GaAs quantum dot lasers by AlGaAs lateral potential barrier layers. Opt Express. 2023; 31:31243–52. 10.1364/OE.498996. 37710648

[102] Qosa H, de Oliveira C, Cizza G et al. Pharmacokinetics, safety, and tolerability of BMS-986263, a lipid nanoparticle containing HSP47 siRNA, in participants with hepatic impairment. Clinical Translational Sci. 2023; 16:1791–802. 10.1111/cts.13581. PMC10582666 37654022

[103] Youssef AS, Ismail M, Han K et al. Population pharmacokinetics of Bepirovirsen in healthy participants and participants with chronic Hepatitis B Virus infection: results from phase 1, 2a, and 2b studies. Infect Dis Ther. 2024; 13:1515–30. 10.1007/s40121-024-00980-9. 38796564 PMC11219612

[104] Xu H, Tong X, Mugundu G et al. Population pharmacokinetic analysis of danvatirsen supporting flat dosing switch. J Pharmacokinet Pharmacodyn. 2019; 46:65–74. 10.1007/s10928-019-09619-6. 30661177

[105] Yu RZ, Collins JW, Hall S et al. Population pharmacokinetic–pharmacodynamic modeling of Inotersen, an antisense oligonucleotide for treatment of patients with hereditary transthyretin amyloidosis. Nucleic Acid Ther. 2020; 30:153–63. 10.1089/nat.2019.0822. 32286934 PMC7249474

[106] Han K, Ito H, Elston R et al. Comparison of pharmacokinetics of the GalNAc-conjugated antisense oligonucleotide GSK3389404 in participants with chronic Hepatitis B infection across the Asia-Pacific Region. Antimicrob Agents Chemother. 2023; 67:e0090022 10.1128/aac.00900-22. 36507675 PMC9872700

[107] Zhang X, Goel V, Attarwala H et al. Patisiran pharmacokinetics, pharmacodynamics, and exposure-response analyses in the phase 3 APOLLO trial in patients with hereditary transthyretin-mediated (hATTR) amyloidosis. J Clin Pharmacol. 2020; 60:37–49. 10.1002/jcph.1480. 31322739 PMC6972979

[108] Li H, Niu X, Zhang Y et al. Pharmacokinetics, safety, and tolerability of the siRNA JNJ-73763989 in healthy Chinese adult participants. Clin Pharmacol Drug Dev. 2023; 12:175–80. 10.1002/cpdd.1197. 36415122

